# The promises, challenges and pathways to room-temperature sodium-sulfur batteries

**DOI:** 10.1093/nsr/nwab050

**Published:** 2021-03-30

**Authors:** Lei Wang, Tao Wang, Lele Peng, Yiliu Wang, Meng Zhang, Jian Zhou, Maoxin Chen, Jinhui Cao, Huilong Fei, Xidong Duan, Jian Zhu, Xiangfeng Duan

**Affiliations:** State Key Laboratory for Chemo/Biosensing and Chemometrics, and College of Chemistry and Chemical Engineering, Hunan Key Laboratory of Two-Dimensional Materials, Hunan University, Changsha410082, China; State Key Laboratory for Chemo/Biosensing and Chemometrics, and College of Chemistry and Chemical Engineering, Hunan Key Laboratory of Two-Dimensional Materials, Hunan University, Changsha410082, China; Department of Chemistry and Biochemistry, University of California, Los Angeles, Los Angeles, CA90095, USA; State Key Laboratory for Chemo/Biosensing and Chemometrics, and College of Chemistry and Chemical Engineering, Hunan Key Laboratory of Two-Dimensional Materials, Hunan University, Changsha410082, China; State Key Laboratory for Chemo/Biosensing and Chemometrics, and College of Chemistry and Chemical Engineering, Hunan Key Laboratory of Two-Dimensional Materials, Hunan University, Changsha410082, China; State Key Laboratory for Chemo/Biosensing and Chemometrics, and College of Chemistry and Chemical Engineering, Hunan Key Laboratory of Two-Dimensional Materials, Hunan University, Changsha410082, China; State Key Laboratory for Chemo/Biosensing and Chemometrics, and College of Chemistry and Chemical Engineering, Hunan Key Laboratory of Two-Dimensional Materials, Hunan University, Changsha410082, China; State Key Laboratory for Chemo/Biosensing and Chemometrics, and College of Chemistry and Chemical Engineering, Hunan Key Laboratory of Two-Dimensional Materials, Hunan University, Changsha410082, China; State Key Laboratory for Chemo/Biosensing and Chemometrics, and College of Chemistry and Chemical Engineering, Hunan Key Laboratory of Two-Dimensional Materials, Hunan University, Changsha410082, China; State Key Laboratory for Chemo/Biosensing and Chemometrics, and College of Chemistry and Chemical Engineering, Hunan Key Laboratory of Two-Dimensional Materials, Hunan University, Changsha410082, China; State Key Laboratory for Chemo/Biosensing and Chemometrics, and College of Chemistry and Chemical Engineering, Hunan Key Laboratory of Two-Dimensional Materials, Hunan University, Changsha410082, China; Department of Chemistry and Biochemistry, University of California, Los Angeles, Los Angeles, CA90095, USA

**Keywords:** room-temperature sodium-sulfur batteries, sulfur cathodes, polysulfide shuttling effects, Na dendrite formation, electrocatalysis

## Abstract

Room-temperature sodium-sulfur batteries (RT-Na-S batteries) are attractive for large-scale energy storage applications owing to their high storage capacity as well as the rich abundance and low cost of the materials. Unfortunately, their practical application is hampered by severe challenges, such as low conductivity of sulfur and its reduced products, volume expansion, polysulfide shuttling effect and Na dendrite formation, which can lead to rapid capacity fading. The review discusses the Na-S-energy-storage chemistry, highlighting its promise, key challenges and potential strategies for large-scale energy storage systems. Specifically, we review the electrochemical principles and the current technical challenges of RT-Na-S batteries, and discuss the strategies to address these obstacles. In particular, we give a comprehensive review of recent progresses in cathodes, anodes, electrolytes, separators and cell configurations, and provide a forward-looking perspective on strategies toward robust high-energy-density RT-Na-S batteries.

## Teaser

Room-temperature sodium-sulfur batteries are attractive for large-scale energy storage applications. This review discusses the Na-S-energy-storage chemistry, highlighting its promise, key challenges and potential strategies, providing a forward-looking perspective toward robust high-energy-density RT-Na-S batteries.

## INTRODUCTION

**Figure 1.**
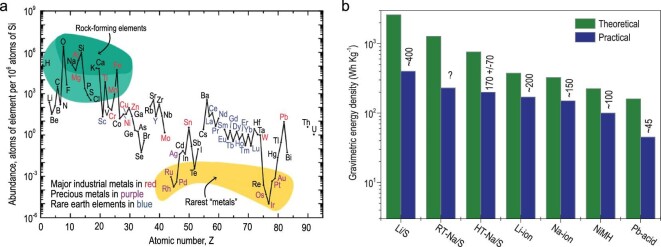
Elemental abundance and potential capacity as a battery material. (a) The abundance of chemical elements in Earth's crust. Adapted with permission from [3]. Copyright 2013, Royal Society of Chemistry. (b) Different battery technologies with theoretical and practical energy densities. Adapted with permission from [6]. Copyright 2015, license Beilstein-Institut.

Lithium-ion battery (LIB) technology has dominated renewable energy supply, particularly for various portable devices (e.g. mobile phones and laptops) and pure electric or hybrid electric vehicles, for its high energy density, stability, long lifespan and reasonable charging time [1]. Nonetheless, the lithium resource shortage (20 ppm in the Earth’s crust) makes LIBs too costly and particularly less practical for grid-level applications [2]. It thus prompts the exploration and development of alternative battery systems built with Earth-abundant materials that are less costly and more environmentally friendly. With this perspective, sodium-sulfur (Na-S) electrochemistry is gaining increasing attention as a promising cost-effective and environmentally benign technology, since both sodium (2.7% in the Earth’s crust) and sulfur (0.048% in the Earth’s crust) are among the most abundant elements on Earth (Fig. 1a) [3].

In fact, the Na-S battery first emerged as a promising energy storage technology over half a century ago, ever since the molten Na-S battery (first-generation Na-S battery) was proposed to operate at high temperatures (>300°C) in the 1960s [4]. Similarly to lithium-sulfur (Li-S) chemistry, Na-S chemistry involves multiple complicated reactions, such as conversion and solid-state diffusion, phase transitions, surface solid electrolyte interface (SEI) film formation and interfacial charge transfer processes [5]. If fully converted into Na_2_S, the theoretical capacity of the Na-S battery is as high as 1675 mAh g^–1^ with regard to the mass loading of sulfur, which is an order of magnitude higher than that of LIBs using insertion-compound cathodes. Meanwhile, the theoretical capacity of the Na anode could reach up to 1166 mAh g^–1^, also considerably higher than the graphite anode in LIBs. Thanks to these merits, Na-S batteries can promise a theoretical specific energy of 1273 kWh kg^–1^, markedly exceeding that of LIBs (350–400 kWh kg^–1^) (Fig. 1b) [6].

However, the first-generation molten Na-S batteries that operate at high temperature always suffer from serious safety challenges due to the melting of ceramic electrolyte NaAl_11_O_17_. Thus, it is of particular interest to lower the operation temperature and enable robust rechargeable Na-S batteries at room-temperature (RT-Na-S batteries, and also second-generation Na-S batteries). However, apart from common problems such as poor conductivity of S and solid-state discharge products, the robust operation of RT-Na-S batteries faces more persistent challenges than Li-S batteries. Firstly, the slow Na^+^ ion diffusion in solid-state ceramic NaAl_11_O_17_ at room temperature rapidly diminishes the rate capability to impractical levels and thus requires the implementation of liquid electrolyte. Secondly, long-chain sodium polysulfides are more soluble in liquid electrolyte than analogous lithium polysulfides [7,8], exacerbating the shuttling effect and capacity fading. Thirdly, the lower reducing capability of sodium ion compared to that of lithium ion leads to more sluggish reaction kinetics between Na and S under a reduced operating temperature. Lastly, the huge volume expansion (260% from S to Na_2_S) could also aggravate structural disintegration and electrical disconnection to compromise the cycling stability. Fortunately, inspired by the recent advancements in Li-S batteries [9], many of these challenges associated with Na-S chemistry may be revisited and mitigated in order to realize robust operation at room temperature.

In this review, we will clarify the basic operating principles and current technical challenges of RT-Na-S batteries. By comprehensively reviewing the recent advancements in cathodes, anodes, electrolytes, separators and cell configurations, we summarize the fundamentals of using various strategies to promote Na-S electrochemistry. Finally, we propose future perspectives, directions and efforts towards practical RT-Na-S batteries (future-generation Na-S batteries), with a particular emphasis on the potential strategies to addressing the critical issues.

## PRINCIPLES OF RT-Na-S BATTERIES

**Figure 2.**
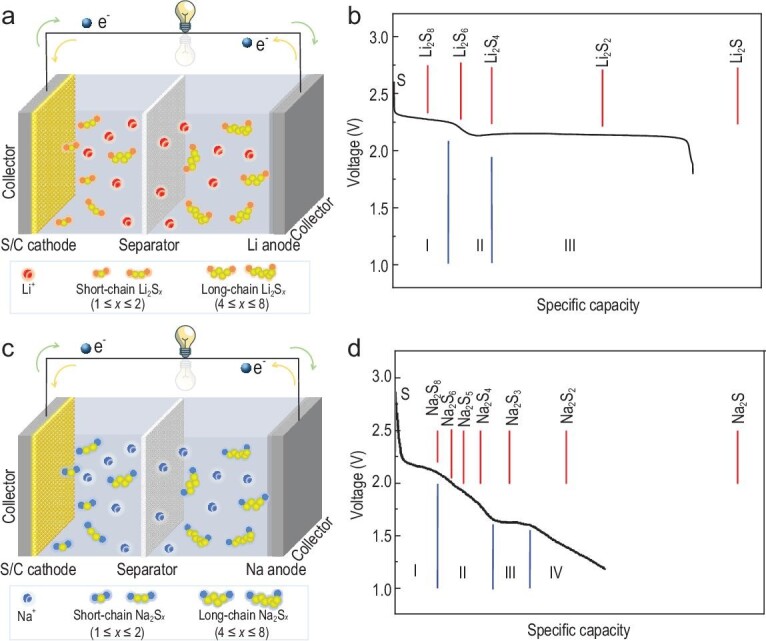
The operating principle of the RT-Na-S battery vs. Li-S battery. (a) Schematic and (b) theoretical vs. practical discharge traits of an Li-S battery. (c) Schematic and (d) theoretical vs. practical discharge traits of an RT-Na-S battery. Adapted with permission from [8]. Copyright 2014, Wiley.

Figure 2 shows the operating principle of the RT-Na-S battery and Li-S battery. The cell configuration of the two types of batteries is almost the same, except for the different anodes (lithium or sodium) and the corresponding electrolytes (Fig. 2a and c). Typically, RT-Na-S batteries are composed of metallic sodium anode, sulfur or sulfur-containing composite cathode, polymer separator, and liquid carbonate-based or ether-based electrolyte [8,10].

The chemistry of Na-S and Li-S is analogous but not identical. Both Li-S and RT-Na-S batteries mainly involve multi-step reactions between the metallic anode and S cathode, as shown by the multi-plateau phenomena typically observed in discharge profile (Fig. 2b and d) [8]. During operation, the ions (Na^+^ or Li^+^) obtained by oxidation of the alkaline metal anode reach the S cathode, producing a series of soluble polysulfide intermediate products (Na_2_S_8_, Na_2_S_6_ and Na_2_S_4_) and insoluble products (Na_2_S_3_ and Na_2_S_2_) before a full conversion to final sulfide (Na_2_S). Based on the fact that there is a two-electron transfer per S molecule, the theoretical specific capacity of S can reach 1675 mAh g^–1^, corresponding to the formation of Li_2_S or Na_2_S.

During the discharge process, the Na metal anode undergoes a simple oxidation reaction to form Na^+^ ions, as shown in the below formulation (1).

(1) \begin{equation*} {\rm{Na\ }} \to {\rm{\ N}}{{\rm{a}}^{\rm{ + }}}{\rm{\ + \ }}{{\rm{e}}^{\rm{ - }}} \end{equation*}

While on the S cathode side, a series of complicated sulfur reduction reactions occur (Fig. 2d). At high-voltage plateau region I (∼2.20 V), a solid-liquid reaction, which produces the highly soluble Na_2_S_8_, occurs following formulation (2).

(2) \begin{equation*} {{\rm{S}}_{\rm{8}}}{\rm{\ + \ 2N}}{{\rm{a}}^{\rm{ + }}}{\rm{\ + \ 2}}{{\rm{e}}^{\rm{ - }}}{\rm{\ }} \to {\rm{\ N}}{{\rm{a}}_{\rm{2}}}{{\rm{S}}_{\rm{8}}} \end{equation*}

Then, the dissolved Na_2_S_8_ is transformed into less soluble Na_2_S_4_ in a sloping region II (2.20–1.65 V), consisting of a liquid-liquid reaction (formulation (3)). At this stage, Na_2_S_6_ and Na_2_S_5_ may be formed through other subtle reactions. Compared with lithium polysulfides (Li_2_S_x_, 4 ≤ x ≤ 8), the generated sodium polysulfides (Na_2_S_x_, 4 ≤ x ≤ 8) are more soluble in the liquid electrolyte, intensifying the shuttling effect.

(3) \begin{equation*} {\rm{N}}{{\rm{a}}_{\rm{2}}}{{\rm{S}}_{\rm{8}}}{\rm{\ + \ 2N}}{{\rm{a}}^{\rm{ + }}}{\rm{\ + \ 2}}{{\rm{e}}^{\rm{ - }}}{\rm{\ }} \to {\rm{\ 2N}}{{\rm{a}}_{\rm{2}}}{{\rm{S}}_{\rm{4}}} \end{equation*}

Region III corresponds to the process in which the dissolved Na_2_S_4_ turns into transitional products of insoluble Na_2_S_3_ or Na_2_S_2_, according to a liquid-solid transition at ≈1.65 V (formulation (4–5)).

(4) \begin{equation*} {\rm{N}}{{\rm{a}}_{\rm{2}}}{{\rm{S}}_{\rm{4}}}{\rm{\ + \ 2/3N}}{{\rm{a}}^{\rm{ + }}}{\rm{\ + \ 2/3}}{{\rm{e}}^{\rm{ - }}}{\rm{\ }} \to {\rm{\ 4/3N}}{{\rm{a}}_{\rm{2}}}{{\rm{S}}_{\rm{3}}} \end{equation*}

(5) \begin{equation*} {\rm{N}}{{\rm{a}}_{\rm{2}}}{{\rm{S}}_{\rm{4}}}{\rm{\ + \ 2N}}{{\rm{a}}^{\rm{ + }}}{\rm{\ + \ 2}}{{\rm{e}}^{\rm{ - }}}{\rm{\ }} \to {\rm{\ 2N}}{{\rm{a}}_{\rm{2}}}{{\rm{S}}_{\rm{2}}} \end{equation*}

Region IV (1.65–1.20 V) corresponds to a solid-solid conversion of insoluble Na_2_S_2_ to Na_2_S (formulation (6)).

(6) \begin{equation*} {\rm{N}}{{\rm{a}}_{\rm{2}}}{{\rm{S}}_{\rm{2}}}{\rm{\ + \ 2N}}{{\rm{a}}^{\rm{ + }}}{\rm{\ + \ 2}}{{\rm{e}}^{\rm{ - }}}{\rm{\ }} \to {\rm{\ 2N}}{{\rm{a}}_{\rm{2}}}{\rm{S}} \end{equation*}

Although the RT-Na-S and Li-S batteries share similar sulfur reduction reaction processes, the actual discharge behaviors show distinct differences, presumably due to the different intrinsic properties of sodium and lithium, such as standard reduction potential (−3.04 V for Li and −2.71 V for Na, vs. standard hydrogen electrode (SHE)), mass and ionic radius, redox ability, etc. Specifically, more active sodium tends to react more with liquid electrolyte solvents and form a less stable SEI, resulting in a larger irreversible capacity. Compared with the Li^+^ ion, the Na^+^ ion has a larger ion radius and less reducing, which means that the transport kinetics are more sluggish, especially in solid-phase conversion. Moreover, discharge products of long-chain sodium polysulfides exhibit more soluble behavior in liquid electrolytes, which is also one of the main factors for the difference between Li-S and Na-S electrochemistry.

## TECHNICAL CHALLENGES

### Poor conductivity

Sulfur itself has a rather low conductivity (≈10^–30^ S cm^–1^), which represents a fundamental challenge that impedes the development of RT-Na-S batteries. Additionally, the reducing products, particularly the solid-state sodium polysulfides (Na_2_S_2_ and Na_2_S), also display very low ionic and electrical conductivity. Moreover, insoluble Na_2_S_2_ and Na_2_S are continuously produced and deposited on the anode and cathode surfaces, gradually forming a thick layer of inactive insoluble agglomerates. This passivation layer causes the gradual loss of active materials and active sites and further interferes with the transfer of ions or electrons, thus resulting in increased cell impedance and degradation of electrode structure. Upon continuous discharge/charge, the final result of these cumulative effects is the rapid fading of capacity over cycling.

### Volume change of sulfur

As is known in the Li-S battery, the large volume difference between S and the fully lithiated Li_2_S stimulates a large strain to destroy the physical integrity of the electrode (electrode pulverization), resulting in a rapid capacity decay [11]. Compared to Li-S batteries, RT-Na-S batteries exhibit a considerably greater volume expansion (260% from S to Na_2_S vs. 80% from S to Li_2_S). Such a dramatic volume change can severely deteriorate the mechanical integrity of the S cathode and compromise the physical and electrical connectivity, leading to additional capacity fading over time. Consequently, the pulverization of the S cathode was considered to be more severe in RT-Na-S batteries compared to Li-S batteries. In this regard, it is essential to fabricate advanced cathode structures to accommodate the volume change of S during deep charge/discharge to improve the cycling stability of RT-Na-S batteries.

### Polysulfide shuttling

A series of intermediate products, including long-chain (Na_2_S*_x_*, 4 ≤ *x* ≤ 8) and short-chain (Na_2_S*_x_*, 1 ≤ *x* ≤ 2) sodium polysulfides, form during the discharging/charging processes. Among them, the long-chain Na_2_S_x_ with high solubility in organic liquid electrolyte can diffuse to the Na anode driven by the concentration gradient, and is reduced to short-chain Na_2_S_x_, which will diffuse back to the cathode and be oxidized to long-chain Na_2_S_x_. This internal cyclical migration phenomenon is called the shuttling effect, which reduces the utilization efficiency of active material and in turn leads to a large loss of its initial capacity. Moreover, metallic sodium with ultra-high reactivity further promotes this phenomenon by rapid reduction of long-chain compounds.

### Formation and growth of Na dendrites

The formation of Na dendrites is due to the uneven nucleation of sodium on the anode. During the electrochemical process, dendrites continue to grow with the cycle and may pierce the separator, causing short circuits inside the battery and inducing safety issues. It is noteworthy that the large difference in atomic and ionic sizes of Na makes it easier for sodium to form unstable electrodeposits and dendrites than lithium [12]. On the other hand, the fresh and reactive sodium metal exposed to the liquid electrolyte might react with the solvent, resulting in non-uniform ionic flux, large Na dendrites and low electrochemical conversion efficiency.

**Figure 3.**
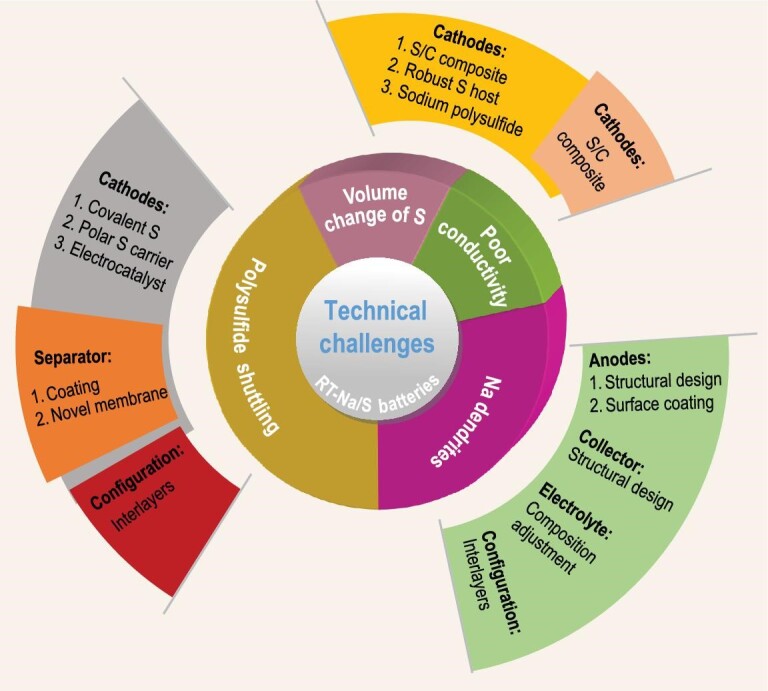
Technical challenges and corresponding strategies for RT-Na-S batteries.

To realize high-performance RT-Na-S batteries, many strategies have been explored in improving the conductivity of the S cathode and reducing products, alleviating the volume change of the S cathode, inhibiting the shuttling effect of sodium polysulfides, and suppressing the growth of Na dendrites. As illustrated in Fig. 3, the optimization of the S cathode by confining sulfur in highly conductive matrices and/or decreasing sulfur particle size, the coating of a protective layer on the Na anode surface, the design of novel current collectors, the adjustment of electrolyte composition, and the assembly of novel cell configurations with interlayers, etc., are proposed as effective solutions. Next, these proposals and related basic principles will be introduced by highlighting some representative examples.

## CATHODES

Widespread application of RT-Na-S batteries is mainly plagued by the challenges of cathode materials, including the insulation of S and its reduced products, as well as the high solubility of intermediate sodium polysulfides (Ns_2_S*_x_*, 4 ≤ *x *≤ 8) in electrolyte. The continuous deposition of insoluble polysulfides on the electrode surface would block the charge transfer, leading to rapid capacity decay. In the past 10 years, numerous efforts have been made to design high-performance cathodes for RT-Na-S batteries. Table 1 summarizes the electrochemical performance of RT-Na-S batteries with various cathodes, mainly including conventional S cathodes, sulfur-containing composite cathodes and sodium polysulfide cathodes.

**Table 1.** Summary of various reported cathodes for RT-Na-S batteries.

Cathode^a^		Electrolyte^a^	Capacity (mAh g^–1^, cycles)	Current (mA g^–1^)	Ref.
S	S/C/PEO	NaCF_3_SO_3_/TEGDME	250 (10)	0.14 mA cm^–2^	[14]
	
Non-covalent S−C	S@OMCS	NaPF_6_/NaNO_3_/TEGDME	300 (1500)	1675	[17]
	S@iMCHS	NaClO_4_/PC/EC	292 (200)	100	[18]
	MCPS	NaClO_4_/SiO_2_-IL-ClO_4_/PC/EC	800 (50)	167.5	[22]
	cZIF-8/S	NaClO_4_/TEGDME	500 (250)	337	[23]
	S/(CNT@MPC)	NaClO_4_/PC/EC	600 (200)	1675	[26]
	CFC/S	NaClO_4_/NaNO_3_/TEGDME	120 (300)	167.5	[27]
	
Covalent S−C	SPAN	NaClO_4_/EC/DMC	500 (18)	0.1 mA cm^–2^	[29]
	c-PANS NFs	NaClO_4_/EC/DEC	153 (500)	250	[31]
	S/PTCDA	NaClO_4_/EC/DMC	400 (150)	150	[32]
	CSCM-18	NaClO_4_/EC/DMC	1000 (900)	80	[33]
	
S-polymer	CS90-rGO	NaClO_4_/NaNO_3_/TEGDME	498 (50)	200	[38]
	S-PETEA	Gel polymer	736 (100)	167.5	[39]
	
S-MXene	S-Ti_3_C_2_T_x_	NaTSFI/PC/EC/FEC	483.7 (600)	1675	[43]
	
Functional group-containing cathode	HSMC-Cu-S	NaClO_4_/EC/DMC	610 (110)	50	[45]
	rGO/S/Mn_x_O_y_	NaClO_4_/NaNO_3_/TEGDME	270 (200)	500	[46]
	
Sodium polysulfide	Na_2_S-MWCNT	NaClO_4_/NaNO_3_/TEGDME	560 (50)	167.5	[47]
	Na_2_S_6_-MWCNT	NaClO_4_/NaNO_3_/TEGDME	400 (30)	No data	[69]

^a^Note: polyethylene oxide (PEO), ordered microporous carbon sphere (OMCS), interconnected mesoporous hollow carbon nanosphere (iMCHS), microporous carbon polyhedron-sulfur composite (MCPS), carbonized zeolitic imidazolate framework-8 (cZIF-8), carbon nanotube (CNT), microporous carbon sheath (MPC), carbon fiber cloth (CFC), polyacrylonitrile (PAN), covalent sulfur-based carbonaceous materials (CSCM), poly(S-pentaerythritol tetraacrylate) (S-PETEA), high-surface-area mesoporous carbon (HSMC), tetraethylene glycol dimethyl ether (TEGDME), propylene carbonate (PC), ethylene carbonate (EC), dimethyl carbonate (DMC), diethyl carbonate (DEC), fluoroethylene carbonate (FEC), sodium bis(trifluoromethylsulfonyl)imide (NaTSFI).

### Conventional sulfur cathode

Sulfur is very difficult to directly use as the cathode and conductive additives are required when preparing electrodes for RT-Na-S batteries. Early research on RT-Na-S battery cathodes can be dated back to 2006. Park *et al.* reported a cathode prepared by ball milling sulfur powder, carbon and polyethylene oxide (PEO) [13]. The S cathode delivered a high initial discharge capacity of 489 mAh g^–1^ (based on S mass) with a well-defined charge/discharge curve, but showed a very poor cycling stability. Later research progress has also demonstrated that high capacity can be obtained by adjusting the proportion of sulfur active material and carbon additives [14–16].

The S cathodes made by conventional approaches are able to exhibit their electrochemical activity, but the overall performance, particularly the cycling, may be unacceptable. This issue can be explained by the following two possible reasons. Firstly, direct physical mixing cannot evenly disperse the sulfur in the electrode, resulting in poor contact between sulfur and conductive additives. Secondly, no restriction for polysulfide shuttling and the agglomeration of sulfur species on the electrode block electron diffusion and transport, thus leading to rapid capacity decline. So, it is necessary to design a novel cathode accompanied by new mechanisms of polysulfide immobilization.

### Sulfur-containing composite cathode

#### Non-covalent sulfur-carbon (S-C) composite cathode

Constructing composite electrodes is considered an effective strategy for improving electrode conductivity, buffering volume change of S, increasing utilization of active S and suppressing the shuttling effect. Carbonaceous materials, such as porous carbon, carbon spheres, graphene and carbon nanotubes (CNTs), can be used as ideal participators due to their high conductivity and chemical stability. Loading sulfur into the carbonaceous matrices by melt diffusion at proper temperature is proven to be a cost-effective and frequent method of constructing the non-covalent S−C composite cathode. The carbonaceous matrices can not only confine the sulfur in the carbonaceous system, but also ensure an effective electrical contact, thus immobilizing the sulfur and improving sulfur utilization. Here, we summarize some non-covalent S—C composites reported in RT-Na-S batteries.

##### Sulfur-porous carbon composite.

Porous carbon materials are good hosts for sulfur loading due to their hierarchical porous structure, which brings about a 3-fold advantage. Firstly, a porous structure can alleviate huge volume expansion of S and prevent soluble polysulfide shuttling. Secondly, a carbon matrix can benefit the charge transfer process during sulfur reduction and oxidation. Moreover, a unique morphological carbonaceous matrix can accommodate and immobilize S. For example, the carbonaceous matrix with larger-size pores can ensure high-content sulfur loading. The smaller-size pores make the carbonaceous matrix capable of reducing the loss of sulfur in the electrochemical process and preventing the shuttling effect of polysulfides.

**Figure 4.**
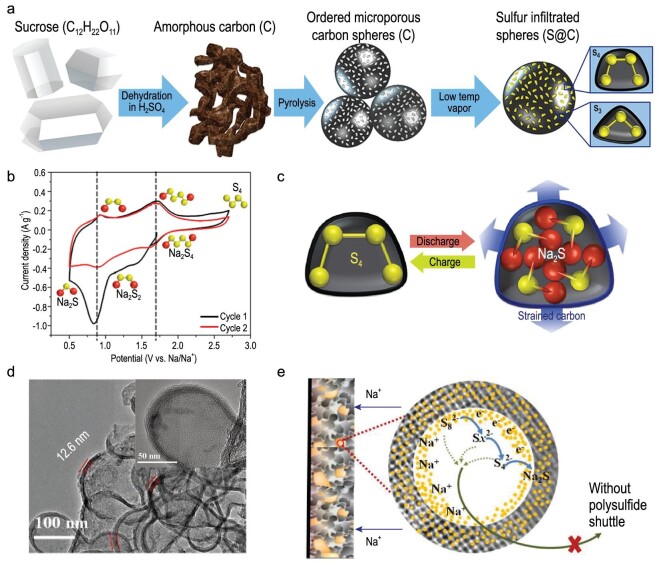
The effects of porous carbon on sulfur and polysulfides. (a) Process for producing microporous cathode materials using sucrose. (b) CV curve of S cathode based on the conversion between the S_2–4_ molecules and S^2–^. (c) Schematic of charge/discharge process involved inside micropores. Adapted with permission from [17]. Copyright 2017, American Chemical Society. (d) TEM images of S@iMCHS nanocomposite and (e) its confinement of sodium polysulfides. Adapted with permission from [18]. Copyright 2016, American Chemical Society.

Carter *et al.* reported a hybrid S cathode by infiltrating sulfur into a microporous carbon template derived from sucrose (Fig. 4a) [17]. The micropores (∼0.5 nm) only allow the formation of small molecule sulfur (S_2–4_), meaning that most conversion occurs between the S_2–4_ molecules and S^2–^ (Fig. 4b), which could eliminate soluble products and prevent the shuttling effect of long-chain polysulfides. From the electrochemical test, the unique electrode exhibited enhanced Coulombic efficiency (>98%) and exceptional durability over 1500 cycles (300 mAh g^–1^ at 1675 mA g^–1^). Such results indicated that the carbon can effectively protect the whole structure from being damaged (Fig. 4c), verifying the important role of a porous carbon host in cycling stability. Similarly, Wang *et al.* took advantage of interconnected mesoporous hollow carbon nanospheres (iMCHSs) in S confinement (Fig. 4d) [18]. The S@iMCHS composite cathode delivered a high reversible capacity; its mesoporous nature could effectively confine the polysulfides and supply open active diffusion channels (Fig. 4e). Gope *et al.* prepared a hierarchical meso/microporous carbon (JNC-1) with a high surface area (>2500 m^2^ g^–1^) via sacrificial-template-assisted synthesis [19]. After being composed with high-content sulfur (>70 wt%), the JNC sulfur composite (JNC-S) exhibited remarkable cycling stability and rate capability. Recently, Guo *et al.* reported an activated ultra-microporous carbon derived from coffee residue as a small S molecules host (ACC-S), which enabled a direct conversion reaction to Na_2_S without polysulfides [20]. As can be seen, the microporous confinement strategy is very effective for ordinary carbon hosts. While the mesopores confine most of the sulfur and polysulfide inside the carbon host, the micropores could regulate the sulfur flux during the charge/discharge cycle.

Metal-organic frameworks (MOFs) are considered to be ideal precursors for the preparation of porous carbon due to their adjustable porous structures with high porosity, multi-dimensional framework and high specific surface area [21]. The carbonized MOF host with microporous structure only provides a path or space for small sulfur molecules to convert between S_4_ and Na_2_S, thereby skipping the formation of long-chain polysulfides and effectively eliminating their shuttling [22]. Moreover, the MOF-derived carbon matrix is always doped by nitrogen, which shows a strong adsorption to sodium polysulfides [23]. Even benefitting from the hierarchical porous structure and heteroatom doping effect, the MOF-derived carbon materials still suffer from low conductivity because of the low degree of graphitization. Therefore, researchers also devoted a great deal of effort to addressing this issue. For instance, Yang *et al.* proposed a 3D railway-like network cathode, where N-doped porous carbon (NPC) could serve as a ‘station’ to host sulfur and the cross-linked CNT networks could serve as a ‘railway’ to facilitate the electron transfer [24]. In addition, the tightly concatenated polyhedrons ensured the integrity of the electrode during the continuous charge/discharge cycle.

In addition to the above advantages in confinement of polysulfides, Ye *et al.* recently proposed a new mechanism based on two-dimensional (2D) Ni-containing MOFs (2D Ni-MOFs) [25]. By adjusting the interaction between polysulfide and MOFs, the dynamic electronic state of the Ni center in a MOF can promote the adsorption and conversion kinetics of polysulfide. Based on the variable structure and controllable composition of MOFs, combined with accurate characterization and theoretical calculation, exploring the involved chemical origins is the key to further developing high-performance S-based cathodes.

##### Sulfur-carbon nanotubes or nanofibers composite.

**Figure 5.**
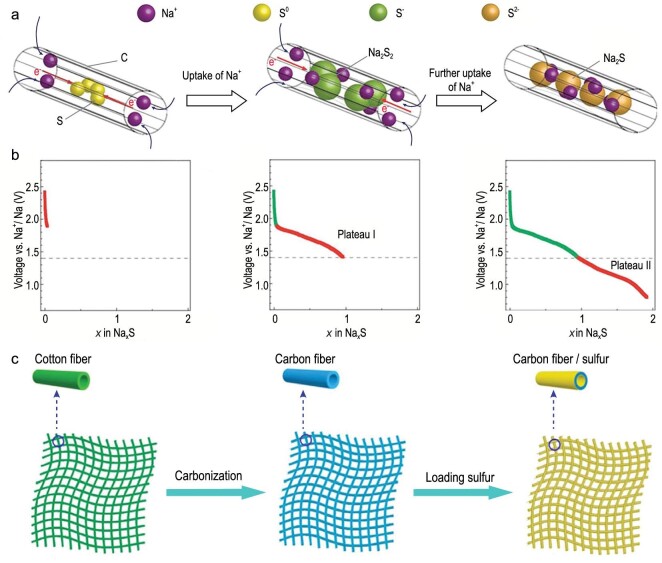
S confinement strategies and electrochemical properties of 1D carbon tubes or fibers. (a) The redox from small S_2–4_ molecules to Na_2_S and (b) the corresponding discharge curves. Adapted with permission from [26]. Copyright 2013, Wiley. (c) Fabrication procedures of preparing CFC/S composite. Adapted with permission from [27]. Copyright 2015, Elsevier.

CNTs and carbon nanofibers (CNFs) with one-dimensional (1D) structures attract much attention due to their unique ion/electron transport channels. For instance, Xin *et al.* reported a coaxial cable-like structure with CNTs inside and an S-containing microporous carbon sheath outside (Fig. 5a) [26]. In the electrochemical process as cathode for RT-Na-S batteries, the electrode underwent two reduction stages: S being reduced to Na_2_S_2_ in Plateau I and to Na_2_S at Plateau II, as per the corresponding discharge/charge curves shown in Fig. 5b. Confined by the micropores, the *in situ* generated S^2–^ is no longer converted into S_8_ molecules but S_2–4_ molecules in the electrochemical process. The electrochemical reaction between small S_2–4_ molecules and S^2–^ avoids the redox of the S_8_ molecule at high potential, skipping the formation of soluble long-chain polysulfides. By effectively preventing the shuttling effect of long-chain polysulfides, this simple and effective strategy to host small sulfur molecules enabled a high specific energy of 750 Wh kg^–1^.

Thanks to the impressive mechanical properties of 1D carbon nanostructures, the sulfur-carbon nanotubes or nanofibers composite also promises application in flexible energy storage, to meet the rapid development of the modern electronics industry, particularly mobile electronics. Lu *et al.* prepared a composite sulfur cathode (CFC/S) with melt-infiltrating sulfur into the carbon fiber cloth (CFC) derived from the carbonized cotton fiber (Fig. 5c) [27]. The 3D interconnected CFC/S composite displays a high absorption capacity of electrolyte, which can confine polysulfides to the cathode area. The free-standing cathode can be used for flexible devices due to its good flexibility and conductivity. Finally, the CFC/S composite delivered a reversible capacity of 390 mAh g^–1^ at 0.1 C and a reversible capacity of 120 mAh g^–1^ after 300 cycles with high area sulfur loading of 2 mg cm^–2^.

**Figure 6.**
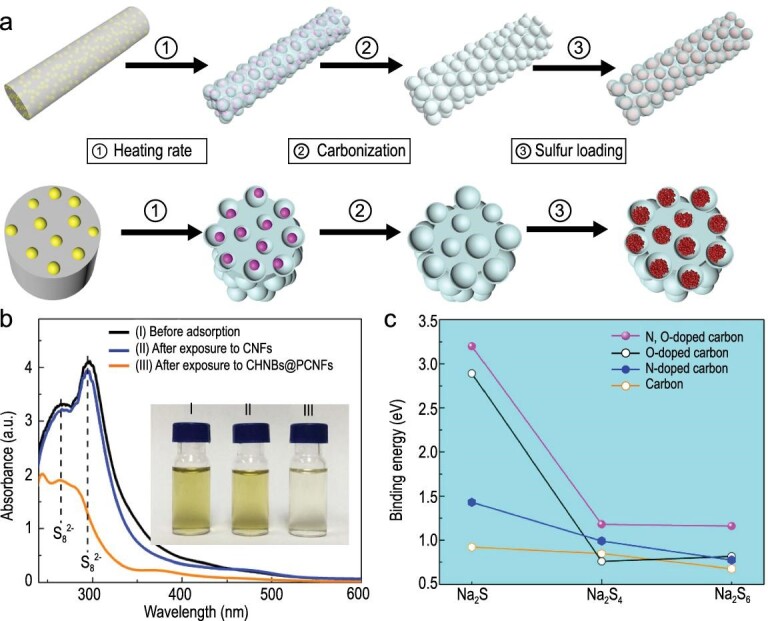
Adsorption of polysulfides by N,O-doped carbon fibers. (a) The preparation of CHNBs@PCNFs. Yellow, pink and red balls represent LiN_3_, Li_3_N and S particles, respectively. (b) UV-vis spectra and corresponding photograph (inset) of the polysulfides adsorption. (c) Comparison of the binding energy between polysulfides and various carbon materials. Adapted with permission from [28]. Copyright 2018, Elsevier.

Recently, Xia *et al.* used the bubbling effect of metal azide (LiN_3_) to prepare carbon hollow nanobubbles on porous CNFs (CHNBs@PCNFs), serving as sulfur carriers (Fig. 6a) [28]. After sulfur loading, the composite cathodes showed improved utilization rate of S, and enhanced cycling stability and rate capability. Such enhancement in overall performance can be attributed to the improved polysulfide adsorption brought by the structural features and the electron-rich heteroatom doping of the carbon hollow nanobubbles. UV-vis spectroscopy and an absorption experiment confirmed that CHNBs@PCNFs exhibit much stronger adsorption capability than CNFs (Fig. 6b). The density functional theory (DFT) calculation regarding the binding energy between Na_2_S, Na_2_S_4_ and Na_2_S_6_ and the doped or non-doped carbons also indicated that the co-doped carbon had a stronger capacity for trapping various polysulfides than single-doped and non-doped carbon, effectively inhibiting the shuttling of polysulfides (Fig. 6c).

#### Covalent sulfur-carbon composite cathode

Some polymers and sulfur can be heated in shielding gas to prepare the carbon sulfur composite with strong C−S bond. In the electrochemical process, the construction of C−S covalent bond can ensure that sulfur exists in the form of small molecule sulfur S_2–4_, which ultimately inhibits the shuttling effect of long-chain sodium polysulfide. In addition, covalent sulfur is highly electrochemically reversible, which is beneficial to cycle stability. Usually, polymers and organic acids have been used as a carbon source, while sulfur powder and sulfur-containing compound (−SO_3_H and/or SO_4_^2–^) have been used as a sulfur source. Also, sulfur-containing polymers as the sulfurizing reagents were selected to generate, *in situ*, covalent C−S composite. We summarized some synthetic strategies for constructing C−S bonds for RT-Na-S batteries by highlighting some typical proposals.

**Figure 7.**
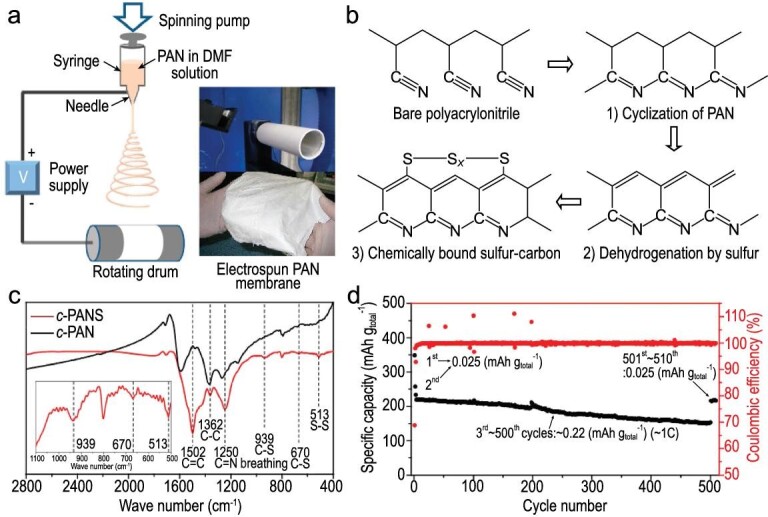
Covalent C-S derived from PAN carbonization and sulfurization. (a) Preparation of PAN membrane and (b) its structural evolution during carbonization and sulfurization. (c) FT-IR spectra of c-PANS and c-PAN. (d) Cycle and coulombic efficiency of c-PANS composite cathode. Adapted with permission from [31]. Copyright 2013, American Chemical Society.

Polyacrylonitrile (PAN), which easily forms 1D fibers, is a commonly used polymer in preparing covalent S-C composite cathodes [29–31]. To investigate the formation mechanism of the C−S bond, Hwang *et al.* prepared PAN nanofibers (NFs) by electrospinning and then incorporated sulfur to obtain S-C composite nanofibers (c-PANS NFs) (Fig. 7a) [31]. During the thermal treatment process, the formation of C−S covalent bonds mainly depends on the three-step reactions (Fig. 7b): cyclization reaction, where nitrile groups (−CN) in PAN are cleaved and bonded to the carbon in the adjacent group; dehydrogenation reaction, where π-conjugated main-chains are produced by reacting with sublimed sulfur; and bonding reaction, where sulfur radicals decomposed by S_8_ molecules react with the carbon matrix. The final formation of C−S covalent bond was confirmed by Raman and Fourier transform infrared (FT-IR) spectra (Fig. 7c). The c-PANS NFs composite cathode delivered a stable cycle with a high average coulombic efficiency of 99.84% (Fig. 7d).

In addition to common PAN, other organics were also used in preparation of covalent S-C composite, for example 3,4,9,10-perylentetracarboxylic dianhydrid (PTCDA) [32] and benzo1,2-b:4,5-b^′^dithiophene-4,8-dione (BDTD) [33], with elemental sulfur as the sulfur source. Recently, sulfurization of benzenedisulfonic acid [34] and phenylphosphinic acid [35] with sulfate (SO_4_^2–^) as the sulfur source has been proved to be an effective strategy in preparing covalent S−C composites with high S content. SO_4_^2–^ can be reduced to S^2–^ during pyrolysis, which forms S-C covalent bonds at certain potential active sites.

**Figure 8.**
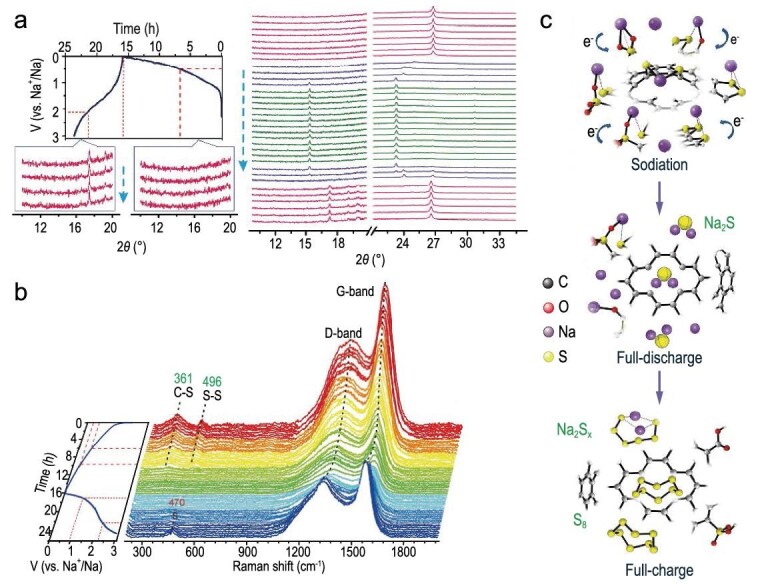
The evolution of covalent C−S bonds during the electrochemical process. (a) *In situ* XRD measurements of the reaction process and (b) *in situ* Raman patterns and the corresponding galvanostatic curve of the HCSC electrode through a discharge/charge cycle. (c) Detailed schematic representation of the first discharge/charge cycle. Adapted with permission from [35]. Copyright 2019, Science China Press and Springer.

Wu *et al.* fabricated a covalent S-C complex with controllable chain length, by converting −SO_3_H and SO_4_^2–^ to short-chain and long-chain C−S, respectively. The resulting complex has a high covalent-sulfur concentration of 40.1 wt% [34]. Another high covalent C−S bridged composite (HCSC) was synthesized by using phenylphosphinic acid and sodium sulfate as precursors [35]. The HCSC has 34.8 wt% covalent sulfur, and the chemical state of covalent-sulfur (C−S−C and/or C−S−S−C) was revealed by Raman, X-ray photoelectron spectroscopy (XPS) analysis and FT-IR spectroscopy. To figure out the bond evolution mechanism during the electrochemical process, Wu *et al.* analyzed the *in situ* synchrotron-based XRD patterns (Fig. 8a) and *in situ* Raman patterns (Fig. 8b). As the XRD patterns show, Na_2_S_x_ and element sulfur were generated after the final desodiation, corresponding to new diffraction peaks at ∼17.5° and ∼19.2°. According to the *in situ* Raman patterns, C−S and S−S bonds broke to form sulfides (Na_2_S) during the discharge process, and then Na_2_S gradually transferred into long-chain polysulfides (Na_2_S_x_) and elemental sulfur after charging to 3.0 V (Fig. 8c). Simultaneously, the I_D_/I_G_ ratio constantly changed as the cycle progressed, which indicated that the carbon skeleton had undergone an irreversible isomerization.

Different from the above synthetic strategy of organic pyrolysis, other new methods for building covalent bonds have also been proposed. For instance, Yan *et al.* prepared, *in situ*, the covalent S-C complex by desulfurization and carbonization in the reaction of carbon disulfide (CS_2_) with a special sulfur-carbon double bond (S=C=S) and red phosphorus [36]. Xiao *et al.* proposed a vapor-infiltration method to achieve covalent C−S in a MOF-derived S,N-doped porous carbon host [37]. Even so, the biggest disadvantage is that these composites suffer relatively low sulfur content, usually less than 40%. How to increase the content of S is of great significance if high-energy RT-Na-S batteries are to satisfy commercial applications.

#### Sulfur-polymer composite cathode

**Figure 9.**
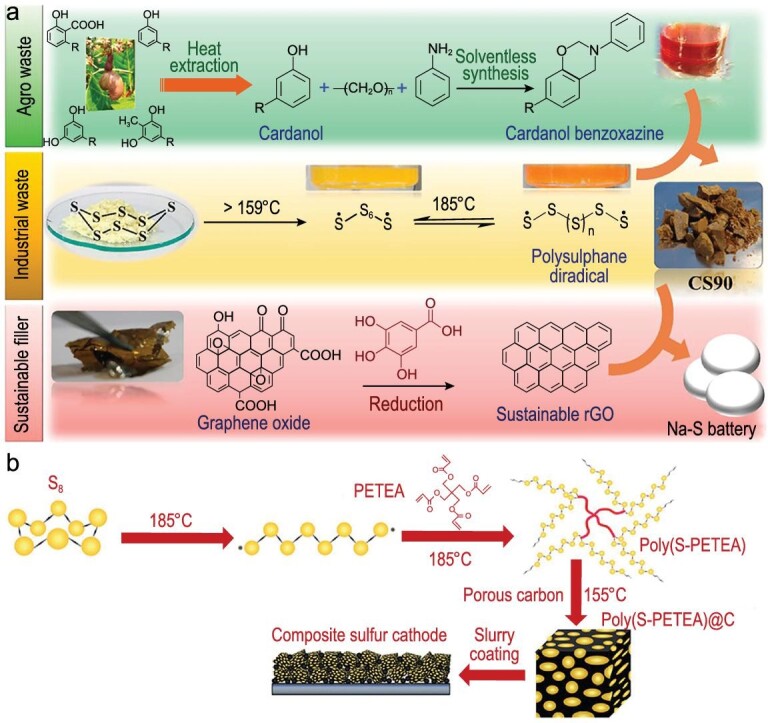
Synthesis strategies of sulfur-containing copolymer. (a) Strategies of preparing the CS90-rGO composite cathode. Adapted with permission from [38]. Copyright 2017, American Chemical Society. (b) The fabrication procedure of poly(S-PETEA)@C cathode. Adapted with permission from [39]. Copyright 2018, Wiley.

Unlike calcinating with the sulfur resource to form a sulfur-carbon mixture, some polymers can be directly composited with sulfur to form sulfur copolymer without high-temperature heating. Ghosh *et al.* declared that the sulfur-embedded polymer (CS90) could be realized via thermal ring-opening polymerization of benzoxazine in the presence of S (Fig. 9a) [38]. It is worth noting that a high content (∼90%) and uniform distribution of S can be achieved by this type of method. After mixing with reduced graphene oxide, the cathode promised high average Coulombic efficiency (99%) and cycle retention, exhibiting a reversible capacity of 650 mAh g^–1^ with a slight capacity fading rate (0.76%). The excellent electrochemical performance of CS90-rGO could be attributed to the ‘plasticizing effect’ of small organosulfide units formed during the electrochemical process. Moreover, Zhou *et al.* prepared a sulfur copolymer via an inverse vulcanization-like process, where poly(S-PETEA) was firstly synthesized by copolymerization of molten S and pentaerythritol tetraacrylate (PETEA), then infiltrated into a mesoporous carbon host (Fig. 9b) [39]. It is worth mentioning that the sulfur content in as-prepared copolymer was up to 97.1 wt%. Although high-content S loading can be achieved through this strategy of forming copolymers, the conductivity of sulfur copolymers is a fatal defect, which always requires additional introduction of conductive carbon materials.

#### Sulfur-MXene composite cathode

2D MXene nanosheets with a formula of M_n+1_X_n_T_x_ (M represents transition metal elements, X represents C and/or N elements, T_x_ can be surface functional groups) have attracted much attention in electrochemical study due to their excellent conductivity and abundant surface/interface properties brought by variable compositional tuning [40]. In the Li-S battery research field, MXene with surface polarity has been verified as capturing lithium polysulfides via Lewis acid interaction [41,42]. Such a unique property can also be applied to the Na-S battery, as it shares the same reaction mechanism with the Li-S battery.

**Figure 10.**
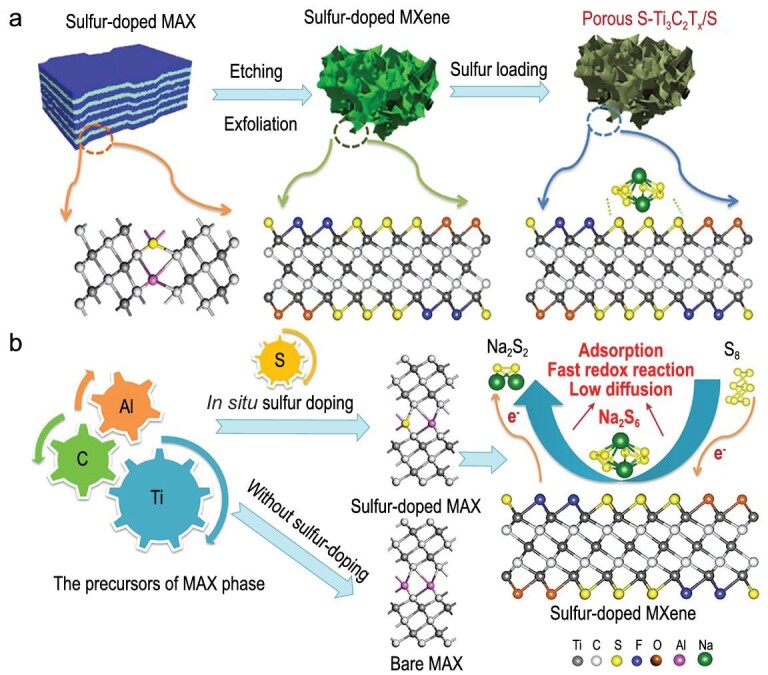
An S-doped MXene (S-Ti_3_C_2_T_x_) as sulfur host and its features. (a) Synthesis of the 3D S-doped MXene/S composite cathode. (b) The multifunction of S-doped MXene. Adapted with permission from [43]. Copyright 2019, American Chemical Society.

3D wrinkled S-doped MXene (S-Ti_3_C_2_T_x_) nanosheets were proposed by Bao *et al.* as a host material for sulfur [43]. After being derived from the precursor (Ti_3_AlC_2_S_x_), S-Ti_3_C_2_T_x_ nanosheets were loaded with S by melt infiltration to prepare the S-Ti_3_C_2_T_x_/S composite cathode, as shown in Fig. 10a. DFT calculations confirmed that S-doped Ti_3_C_2_T_x_ has a strong chemical adsorption for sodium polysulfide, which can efficiently capture sodium polysulfide and inhibit its diffusion (Fig. 10b). The S-Ti_3_C_2_T_x_/S could deliver a high reversible capacity of 577 mAh g^–1^ at 2 C even after 500 cycles. However, the role of surface functional groups that directly relate to polarity needs to be further refined, and exploring other functions besides adsorption is also a meaningful work.

#### Functional group-containing sulfur composite cathode

##### Sulfiphilic group-containing cathodes.

In recent years, nanostructured transition metal oxides with a controllable ‘sulfiphilic surface’ were considered to be capable of anchoring polysulfides for Li-S batteries [44]. The polar host oxides can efficiently adsorb polysulfides during discharge processes, which mitigates the polysulfide shuttling effect. The similar features between Na and Li mean this strategy can be transferred to fabricate RT-Na-S battery cathodes.

Zheng *et al.* reported a nano-copper-assisted method to immobilize sulfur in a high-surface-area mesoporous carbon (HSMC-Cu-S) cathode with S content of 50% for RT-Na-S batteries [45]. The incorporated copper nanoparticles show a strong interaction with polysulfides, and can significantly enhance the electrical conductivity of the entire electrode, even with a small amount of 10 wt%. As a result, the HSMC-Cu-S composite displayed a high initial capacity (∼1000 mAh g^–1^) and a considerably enhanced cycling stability with 86% retention rate.

**Figure 11.**
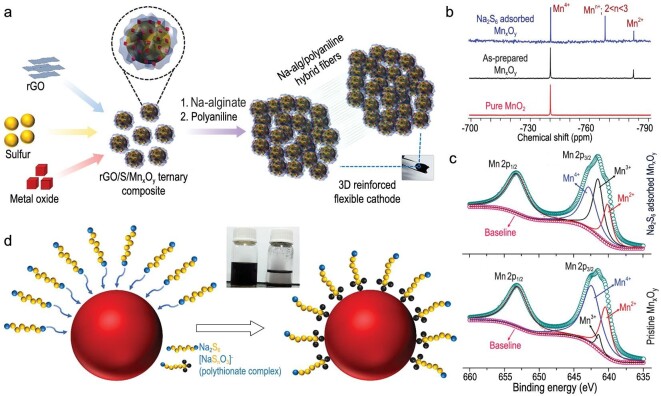
The function of metal oxide (Mn_x_O_y_) in anchoring polysulfides. (a) Illustration of the freestanding rGO/S/Mn_x_O_y_ composite. (b) Solid-state Mn NMR spectra of Mn_x_O_y_ at different states. (c) Mn 2p XPS spectra of as-prepared and Na_2_S_6_ adsorbed Mn_x_O_y_. (d) Potential redox reactions on the surface of Mn_x_O_y_. Adapted with permission from [46]. Copyright 2019, American Chemical Society.

Based on the experience of suppressing polysulfide shuttling by mixed-valence manganese oxide in Li-S batteries, Ghosh *et al.* proposed a unique hybrid cathode combining reduced graphene oxides with excellent electrical conductivity, mixed-valence manganese oxides with polysulfide adsorption ability, and sulfur nanoparticles [46]. As illustrated in Fig. 11a, Na alginate/polyaniline hybrid matrix could ensure the electrode integrity after incorporation of Mn_x_O_y_ nanocrystals into rGO. The interaction between Mn_x_O_y_ and intermediate polysulfides was explained by solid-state nuclear magnetic resonance (NMR) and XPS analysis. The slight downfield shifting of peaks revealed a weak dipole–dipole interaction between the two polar materials, while a new peak emerged, indicating the redox between Na_2_S_6_ and Mn_x_O_y_ (Fig. 11b), which was also confirmed by the XPS results in Fig. 11c. Along with these analyses, the surface reaction mechanism was outlined in Fig. 11d, where manganese oxide oxidized sodium polysulfides to sodium polythionates.

##### Electrocatalyst-containing cathodes.

Polysulfide's shuttling effect still obstructs the development of sulfur-based batteries, even if considerable strategies have been developed to address this issue. Introducing a catalyst into the sulfur-based system was considered to be an effective strategy in accelerating the conversion of polysulfide compounds and ultimately achieving exceptional performance [47–49]. Such catalyst systems can include metals, metal oxides/sulfides/nitrides/carbides, single atoms, organic molecules, etc. In particular, atomic-scale metal materials with amazing electronic properties, maximum atomic utilization and outstanding electrocatalytic activity received extensive attention.

**Figure 12.**
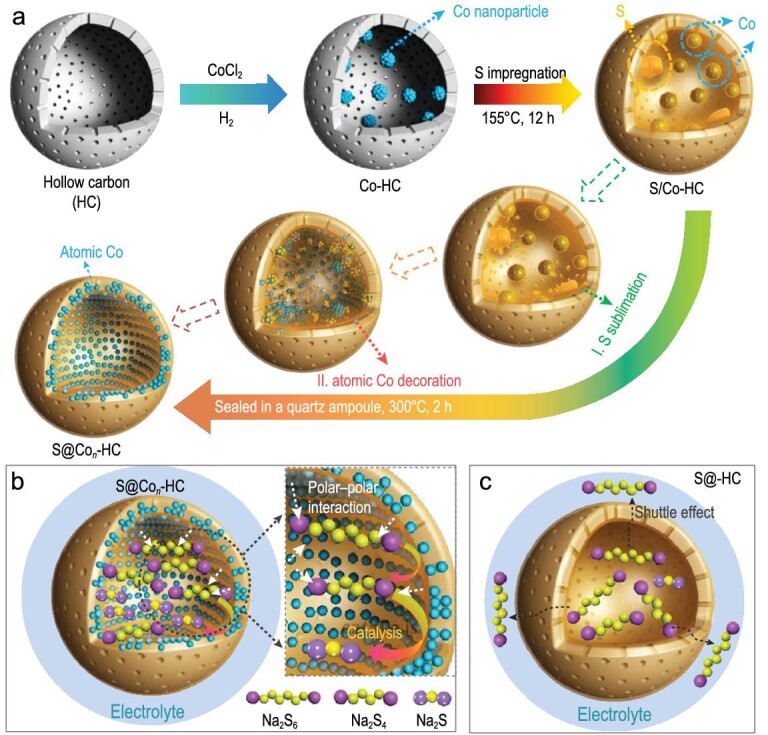
Atomic Co catalyst promoting Na-S chemistry. (a) Schematic illustration of synthesis of S@Co_n_-HC. (b and c) The distinction in electrode reaction mechanism of S@Co_n_-HC and S@HC, respectively. Adapted with permission from [50]. Copyright 2018, Nature Publishing Group.

Recently, Zhang *et al.* reported atomic Co as an efficient electrocatalyst in the S cathode for advanced RT-Na-S batteries [50]. Sulfur was impregnated into atomic Co-decorated hollow carbon nanospheres (S@Co_n_-HC), which were synthesized by multi-step reactions, including the initial preparation of Co-decorated HC and the subsequent redispersion of Co into the carbon shell driven by S molecule diffusion (Fig. 12a). Hollow carbon spheres with micropores finally successfully encapsulated Co nanoparticles (∼3 nm) and sulfur. The S@Co_n_-HC cathode shows superior durability, indicating that the hollow carbon host can confine sodium polysulfides and effectively control its harmful dissolution. More importantly, differently to the traditional S-loaded hollow carbon nanospheres (S@HC), the modification of the atomic Co in S@Co_n_-HC can also provide electrocatalytic

 

capacity for the S cathode, improving conductivity and electrochemical activity, as shown in Fig. 12b and c. From the results of *in situ* synchrotron XRD, no diffraction peaks of Na_2_S_2_ were found, which indicated the reaction from Na_2_S_4_ to Na_2_S was accelerated by the atomic Co catalyst. Importantly, this proposal opens a new exploration path to ameliorate the electrochemical performance of S cathodes.

**Figure 13.**
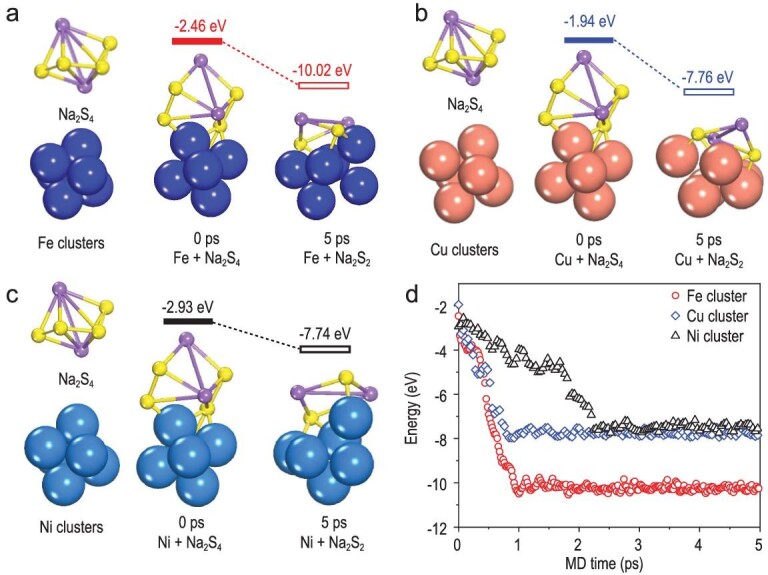
Comparison of polysulfides adsorption by different transition metal nanoclusters. (a–c) Na_2_S_4_ on Fe_6_, Cu_6_ and Ni_6_ nanoclusters in the initial state at 0 ps, and at 5 ps. (d) Adsorption energies (eV) of Na_2_S_4_ on metal nanoclusters as a function of *ab initio* molecular dynamics simulation time (fs). Adapted with permission from [51]. Copyright 2019, Wiley.

Transition-metal (Fe, Cu and Ni) nanoclusters on hollow carbon nanospheres (S@M-HC) were designed as S hosts for RT-Na-S batteries by Zhang and co-workers [51]. The XPS results indicated the formation of Fe/Cu/Ni-S chemical bonds between nanoclusters and S. Among these, the Fe nanocluster-based cathode delivered the greatest reversible capacity. In order to clarify the relationship between the nanocluster type and catalytic properties, they performed DFT calculations using *ab initio* molecular dynamics (AIMD) simulations (Fig. 13). After adsorbing on Fe, Cu and Ni nanoclusters/carbon, the structural evolution of Na_2_S_4_ and corresponding adsorption energies were illustrated in Fig. 13a–c and Fig. 13d, respectively. Obviously, the interaction between Na_2_S_4_ and Fe nanoclusters with greater adsorption energy was stronger than that of Cu and Ni, which implied Fe nanoclusters possess higher catalytic activity and finally suppress the shuttling effect.

In addition to transition-metal particles or nanoclusters [50–53], noble metals like Au are also used in electrocatalyzing the low-kinetics conversion of polysulfides into Na_2_S [54]. Due to the O^2–^ state of the oxygen, metal oxides are considered to be an effective catalyst through strong interaction with polysulfides. Xu *et al.* reported the electrocatalysis effect of the VO_2_/rGO catalyst on rapidly converting long-chain polysulfides to Na_2_S_2_ and/or Na_2_S, which ultimately improved the durability of Na-S batteries [55]. Metal sulfides with sulfiphilic sites can also strongly interact with and catalyze sodium polysulfides [56–59]. Dou’s group rationally designed various sulfides (such as NiS_2_ [56], FeS_2_ [57], ZnS and CoS_2_ [58]) in N-doped porous carbon for high immobilization and conversion of polysulfides. Combined with DFT calculation, they proposed the N atoms and NiS_2_ nanocrystals to be electrocatalytic sites, where rapid conversion from polysulfide to Na_2_S occurred. Xu’s group investigated the polar and catalytic properties of cobalt chalcogenide (CoS_2_, CoSe_2_ and CoTe_2_) as the host of sodium polysulfide, and proposed that these properties should be attributed to structural differences rather than the types of anions (i.e. S, Se and Te) [60]. In addition, carbide (Co_3_C [61], VC [62]) and phosphide (CoP) [63] have also been proposed as electrocatalysts to accelerate the conversion reaction. Interestingly, Kumar *et al.* described a new conceptual mechanism using a highly activated carbon cloth (ACC) as a sulfiphilic host [64]. The ACC catalyzes the redox conversion between intermediate polysulfide radical monoanions (S_3_^•–^) and short-chain polysulfides (S_2_^2–^) through a free-radical mechanism.

So far, proposals of suitable electrocatalysts for promoting the conversion of sodium polysulfides are limited. The related ‘catalysis-conversion’ mechanism still needs to be refined and clarified, eliminating the role of catalyst carriers introduced at the same time and the reaction of the catalyst itself with alkali sodium or sodium ions. Very recently, Peng *et al.* proposed a fundamental look at electrocatalysts for Li-S batteries, using a nitrogen- and sulfur-doped holey graphene framework as a model [65]. Edge carbon atoms adjacent to the heteroatoms serve as catalytic centers, while dual-doping tunes the *p*-band center of the active carbon atoms to achieve an optimal lithium polysulfides radical adsorption, minimizing the overpotential. This fundamental proposal will provide a significant reference for further development of electrocatalyst-containing cathodes in RT-Na-S batteries.

### Sodium polysulfide cathodes

In general, sodium-free cathodes in RT-Na-S batteries, such as pure S or S−C composite, are required to be matched with metallic Na anodes. Consequently, the formation and growth of sodium dendrites would definitely cause huge damage to the cell. In addition, S cathodes would undergo a huge volume change accompanied by the deep sodiation process, undermining the integrity of the electrode. These factors unavoidably lead to low Coulombic efficiency, irreversible loss of active species and decay of battery life and even safety. Thus, some research efforts regarding short/long-chain sodium polysulfides as alternate cathodes have been proposed to alleviate the issues of pure sulfur and sodium metal to some extent. We highlight a few reports on sodium polysulfide cathodes below.

#### Short-chain sodium sulfide cathode

Yu *et al.* reported the short-chain Na_2_S/multi-walled carbon nanotube (MWCNT) composite material as a cathode for RT-Na-S batteries [66]. The MWCNT fabric can ensure efficient electron conduction, accelerate ion transport and trap charged/discharged products during electrochemical cycling. As a result, the Na_2_S-MWCNT cathode revealed clear charge/discharge curves and delivered a high energy density of ∼250 Wh kg^–1^ (based on total loading of the electrode) with a high Coulombic efficiency (>90%). However, the ability to withstand long cycles is limited. Later, they followed the concept above by employing Na_2_S as the cathode, but replaced expensive MWCNT with activated CNFs [67]. The activated CNFs with self-weaving property were used as the matrix for Na_2_S to form a free-standing cathode.

To further solve its low ionic and electrical conductivities, hollow Na_2_S nanospheres embedded in a 3D sponge-like carbon frame were synthesized by Wang and co-workers [68]. The nano-sized hollow architecture of Na_2_S played a key role in fast ion/electron transport, which resulted in efficient utilization of Na_2_S, and enhanced reaction kinetics. Moreover, the porous carbon matrix can act as a maze for trapping long-chain sodium polysulfides, which could alleviate the shuttling effect. Based on excellent structural characteristics, the frogspawn-coral-structured Na_2_S/carbon composite delivered a superior high-rate performance. Combined with Na-metal free anode (Sn@C), the full batteries showed exceptional cycling performance.

#### Long-chain sodium polysulfide cathodes

**Figure 14.**
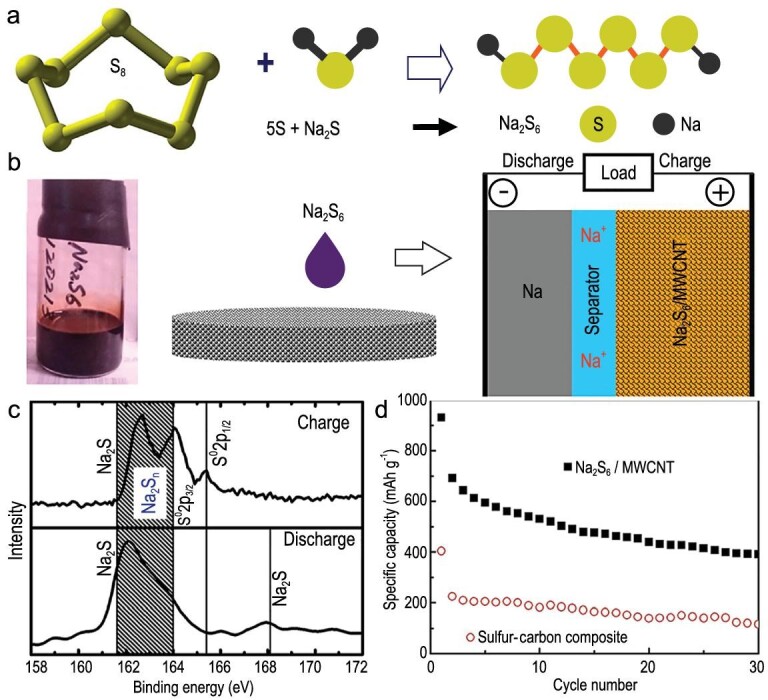
Electrochemical behaviors of a Na_2_S_6_-based composite cathode (Na_2_S_6_/MWCNT). (a) Schematic of the Na_2_S_6_ preparation process. (b) Schematic of the preparation of the Na||N_2_S_6_/MWCNT cell. (c) XPS spectrum of S 2p in the cathode upon initial full charge/discharge. (d) Cycling performances of the Na_2_S_6_/MWCNT cathode and the conventional S/C cathode. Adapted with permission from [69]. Copyright 2015, Wiley.

Yu and co-workers stoichiometrically mixed S powder and Na_2_S into a blank electrolyte containing NaClO_4_ and NaNO_3_ in the tetraethylene glycol dimethyl ether (TEGDME) solvent to prepare a dissolved Na_2_S_6_ catholyte (Fig. 14a) [69]. The free-standing Na_2_S_6_ and multi-wall carbon nanotube (Na_2_S_6_-MWCNT) composite cathode was then prepared by adding sodium polysulfide catholyte into the MWCNT fabric (Fig. 14b). To identify the products' compositions during the electrochemical process, the XPS analysis (Fig. 14c) revealed that the peaks of elemental sulfur and long-chain polysulfides appeared after charging, while the characteristic peaks of Na_2_S or short-chain polysulfide species were detected after initial full discharging. The shifts of polysulfide peak to lower-binding-energy positions in shadow areas indicated that most of the long-chain sodium polysulfides were converted into short-chain polysulfide and a small amount of Na_2_S during discharge. Unfortunately, the capacity fading was obvious (Fig. 14d), which may be attributed to the incomplete reversibility of the transition of short-chain sodium polysulfides and/or Na_2_S during cycling.

**Figure 15.**
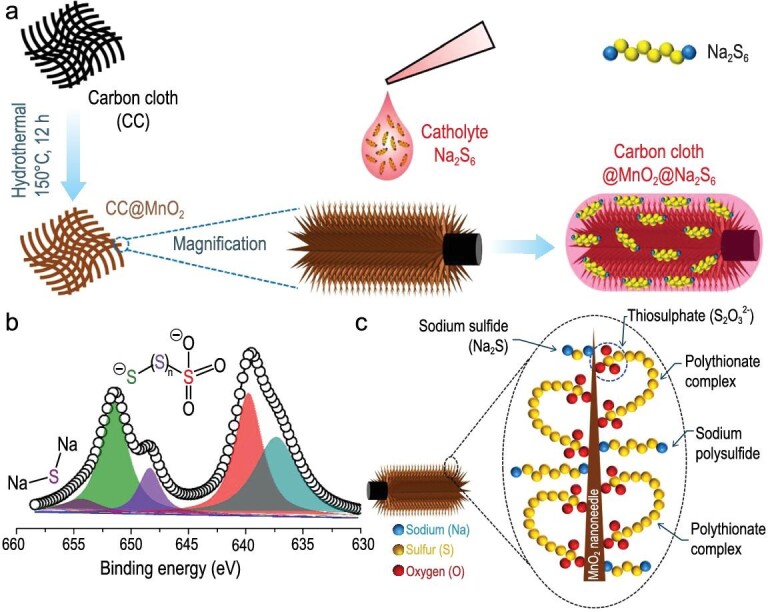
Preparation and superiority of a Na_2_S_6_-based composite cathode (Na_2_S_6_/CC@MnO_2_). (a) The preparation of CC@MnO_2_@Na_2_S_6_ cathode. (b) S 2p_3/2_ spectrum of CC@MnO_2_@Na_2_S_6_. (c) Schematic representation of the possible interactions of manganese oxide with different sodium polysulfides and polythionate complexes. Adapted with permission from [70]. Copyright 2019, American Chemical Society.

Afterwards, Kumar *et al.* directly incorporated liquid-phase Na_2_S_6_ into the flexible conductive substrate as a cathode for RT-Na-S batteries (Fig. 15a) [70]. The liquid-phase catholyte ensured the homogeneous distribution on the matrix of MnO_2_ nanoneedle arrays on carbon cloth (CC@MnO_2_), which enhanced the utilization of sulfur. The CC@MnO_2_ matrix with sulfiphilic property can easily capture sodium polysulfides to inhibit the shuttling effect. MnO_2_ played a key role as a polysulfide reservoir, which was investigated by XPS. The S 2p_3/2_ spectrum of the composite cathode in Fig. 15b proved the formation of polythionate complexes containing thiosulfate (S_2_O_3_^2–^) group and Na_2_S with the reduction of manganese, which is clearly depicted in Fig. 15c. The proposal of this strategy can provide a reference for the exploration of a new type of cathode.

## ANODES

The much richer abundance of sodium in Earth's crust is the biggest advantage over lithium, and makes it a promising alternative anode material. In addition, the low electrochemical reduction potential (−2.71 V vs. SHE) of sodium can ensure that the voltage of a cell composed of a Na metal anode and a suitable cathode is greater than 2 V, and the corresponding device shows a reasonable energy density. Moreover, the extremely high theoretical capacity (1166 mAh g^–1^) of Na has prompted the adoption of metallic Na as an anode. However, sodium unevenly nucleates on the anode surface during the charge/discharge process, resulting in the formation of sodium dendrites, which may pierce the separator and cause a short circuit inside the battery. To suppress the growth of sodium dendrites, many strategies have been proposed, such as constructing various structures of metallic sodium, coating artificial protective layers on sodium, optimizing electrolyte components or additives, and designing an unconventional collector [71]. In this section, we focus on the treatment of sodium metal. The contents of the electrolyte and current collector will be described below.

### Structural design

From a safety point of view, it is necessary to develop suitable anode materials to replace sodium metal. But from the perspective of energy/cost ratio, Na is an attractive option due to its abundance and low price. This is especially true when combined with a high-capacity and low-cost S cathode. Therefore, developing different architectures of sodium metal is a viable strategy.

Hu’s group encapsulated sodium metal into porous carbonized wood to form a composite electrode (Na-wood), which solves almost all the aforementioned problems [72]. In the long-term cycle, the Na plating/stripping is easily controlled by the porous channel to avoid uncontrolled Na deposition. Further, the high conductive surface area will effectively distribute current to avoid uneven nucleation of Na caused by excessive local current density. Thanks to the unique structural properties, the Na-wood composite cathode delivered superior cycle ability over 500 h at 1.0 mA cm^–2^ with a low overpotential of 30 mV at 0.5 mA cm^–2^. Research on the nanostructured carrier of the metal sodium anode is still in the initial stage. At the same time, it is necessary to use advanced technologies to fundamentally understand the growth mechanisms of Na dendrites.

### Coating artificial protective layers

For Li metal batteries, an *ex situ* coat of protective layers is effective in reducing dendrite growth. However, due to the high activity and low melting point of Na metal, the strategies which can successfully deposit a conformable protective coating on its surface are limited. Among them, atomic layer deposition (ALD), as an excellent technology, can achieve deposition at the nanoscale level with high coverage at low temperatures, and maintain the stability of the substrate morphologies. Al_2_O_3_ was successfully coated on the sodium metal anode by ALD, reported by Luo [73] and Zhao [74]. Compared with bare Na foil, ultra-thin Al_2_O_3_ layer coated Na foil without dendrites showed enhanced Na plating/stripping performance. Also, Kim *et al.* stabilized the metallic Na anode by coating a free-standing inorganic/organic composite protective layer (FCPL) on the surface, which is composed of Al_2_O_3_ inorganic particles and poly(vinylidene fluoride-co-hexafluoropropylene) (PVDF-HFP) polymers [75].

**Figure 16.**
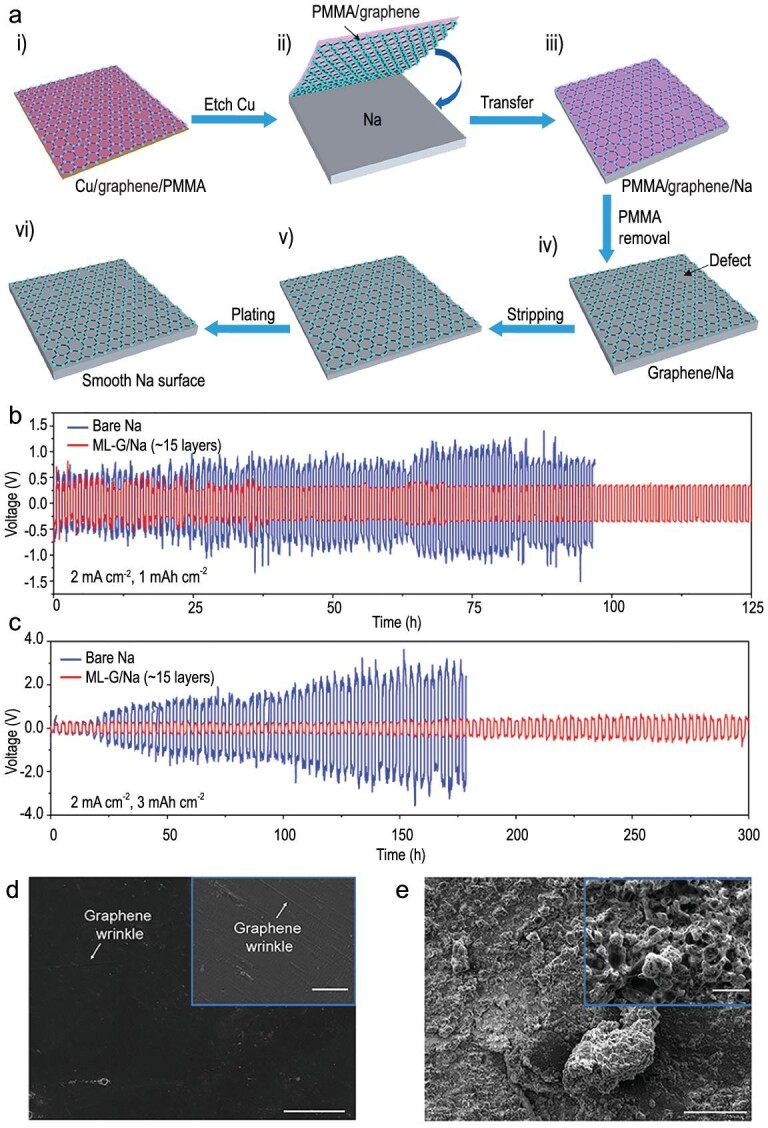
Suppression of Na dendrites by graphene surface coating. (a) The transfer process of graphene film (i–iii) and Na stripping/plating (iv–vi). (b) and (c) Cycle retentions of ML/G-Na electrodes (red) and bare Na (blue) with a cycling capacity of 1 and 3 mAh cm^–2^. (d) and (e) SEM images of ML-G/Na and bare Na electrode surface after 100 cycles (scale bars, 200 μm; scale bars of insets, 10 μm). Adapted with permission from [76]. Copyright 2017, American Chemical Society.

Unlike the ALD treatment, a free-standing graphene film with adjustable thickness was applied on metallic sodium surface by the process illustrated in Fig. 16a [76]. When using a multi-layer graphene film (ML-G) with a thickness of ∼5 nm as the coating layer, the ML/G-Na electrodes exhibited good stability at a current density of 2 mA cm^–2^ with a high cycling capacity of 3 mAh cm^–2^ (Fig. 16b and c). Comparing the surface morphologies of coated and bare Na electrodes after long cycles (Fig. 16d and e), it can be seen that a smooth surface without random Na depositions was found on the ML-G/Na electrode while a rough surface with Na dendrites was on the bare Na electrode. Recently, Kumar *et al.* constructed an artificial metal-alloy interphase (Na_x_Sn_y_) on the metallic Na surface by a solid-vapor reaction [77]. The interphase with strong adhesion, high ionic conductivity, high Young's modulus and low electrolyte permeability realized reversible and long-term deposition of sodium.

Uniform Na plating is necessary for a stable and robust sodium-metal battery. More inhibition strategies need to be borrowed from the proposals on suppressing lithium dendrite. Simultaneously, the difference between Na and Li in size, ionicity and reactivity still needs to be taken into account. In addition, stable liquid–metal interfaces, such as the growth of SEI, also affect the electrodeposition of sodium metal anodes and cell lifespan. More detailed and systematic research will definitely lead the development of sodium metal-based batteries, including RT-Na-S batteries.

## ELECTROLYTE

Electrolytes afford ion transport pathways between the Na anode and S cathode, directly determining the performance of batteries. Generally, two types of electrolytes are most commonly used, including organic liquid electrolyte and solid-state electrolyte. Solid-state electrolyte can potentially avoid issues in organic liquid electrolytes, such as leakage, the dissolution of polysulfides and the shuttling effect. However, poor conductivity, poor interface stability and high working temperature affect the practical application of solid-state electrolyte. Liquid electrolyte with good ionic conductivity is more conducive to forming a uniform current flux at the interface, inhibiting the formation of Na dendrites. However, general liquid electrolyte can severely dissolve the intermediate polysulfides, leading to poor Coulomb efficiency and cyclic stability. Thus, reasonable selection of an electrolyte with optimal composition is essential to enhance the electrochemical properties of RT-Na-S batteries.

### Solid-state electrolyte

Solid-state electrolytes present more effective restriction for the shuttling effect of sodium polysulfides than liquid electrolytes. In addition, active sodium is more prone to harmful reactions with liquid electrolyte in RT-Na-S batteries. These will stimulate solid-state electrolytes to replace the common liquid electrolytes.

Due to the high ionic conductivity at room temperature, NASICON-type solid-state electrolyte has been used as a solid electrolyte for RT-Na-S batteries. However, the charge transfer barriers at the solid–solid interface still exist and continue to restrict the performance of solid-state Na-S batteries. To optimize the interface impedance, Manthiram’s group proposed a solid-state electrolyte of NASICON-type Na_3_Zr_2_Si_2_PO_12_@ PIN, coated by a polymer layer with intrinsic nanoporosity [78]. As another example, An *et al.* recently enhanced interfacial stability by using an ionic liquid *N*-butyl-N-methylpyrrolidinium bis(fluorosulfonyl)imide (Pyr_14_FSI) between the NaSn alloy anode and Na_3_PS_4_ solid electrolyte [79].

In addition, non-aqueous polymer-based gels have also been studied as electrolyte for RT-Na-S batteries, such as NaCF_3_SO_3_-PEO [80], NaCF_3_SO_3_-polyvinylidene fluoride (PVDF)-tetraglyme [13], NaCF_3_SO_3_ PVDF-hexafluoropropylene (HFP)-tetraglyme [15] and NaCF_3_SO_3_/SiO_2_-ethylene carbonate (EC)-propylene carbonate (PC)-(PVDFHFP) [81], PEO-NaFSI-TiO_2_ [82]. However, the low ionic conductivity, poor dimensional stability and poor interfacial stability of the polymer electrolyte lead to poor cycling stability. Therefore, for basic research and even industrial production, these disadvantageous factors must be eliminated.

### Organic liquid electrolytes

Drawing lessons from decades of in-depth research on lithium metal anodes, ordinary liquid electrolytes cannot form a uniform SEI on the surface of alkali metals. The exposed fresh metal Na reacts irreversibly with the electrolyte solvent, resulting in low Coulombic efficiency. At the same time, uneven ion flux will also cause dendrites to grow. Regulating electrolyte components or adding additives, as well as developing new electrolytes are considered to be effective strategies.

#### Carbonate-based electrolyte

Because of its favorable anode passivation, high ionic conductivity and good electrochemical stability, numerous efforts have been aimed at developing carbonate-based electrolyte. EC, DMC, DEC and PC are the most common organic solvents with sodium-containing salt (NaClO_4_ and NaPF_6_). Based on the experience with the Li-S battery, carbonate-based electrolyte can be competent when S is perfectly confined to porous carbon or polymers through encapsulation or covalent bonding. However, during the initial discharge process, a potential nucleophilic reaction may occur between carbonate and reduced-solubility polysulfide, resulting in the loss of electrolyte and active materials, which ultimately leads to rapid capacity degradation. In addition, these conventional electrolytes cannot meet the requirements of stable SEI film and no dendrite formation during Na plating/stripping, and the target of inhibiting the shuttling of polysulfides during S redox.

**Figure 17.**
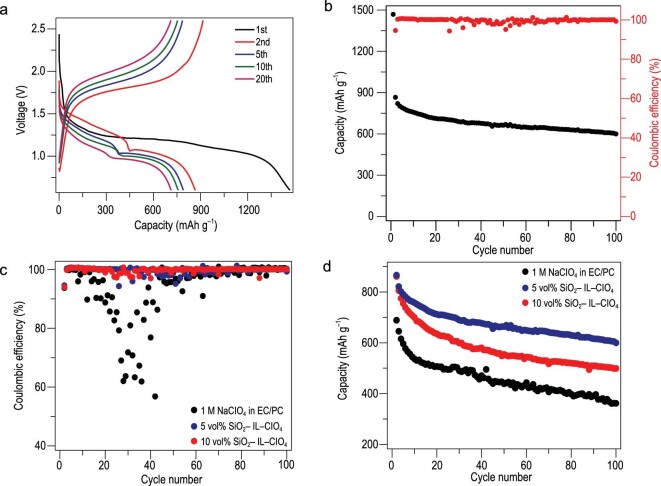
Use of an additive (SiO_2_-IL-ClO_4_) in normal carbonate electrolyte. (a) The discharge/charge curves and (b) cycling with Coulombic efficiency of the cell with 5 vol% of SiO_2_-IL-ClO_4_. The comparison in (c) Coulombic efficiencies and (d) specific capacities for the cell containing different contents of SiO_2_-IL-ClO_4_ at 0.5 C. Adapted with permission from [22]. Copyright 2016, Nature Publishing Group.

An additive of ionic liquid 1-methyl-3-propylimidazolium-chlorate tethered to SiO_2_ nanoparticles (SiO_2_-IL-ClO_4_) in common carbonate-based electrolyte (NaClO_4_ in EC/PC) was reported by Wei *et al.* With the addition of 5 vol% of SiO_2_-IL-ClO_4_, a high reversible capacity of 600 mAh g^–1^ was achieved with nearly 100% Coulombic efficiency at current density of 0.5 C (Fig. 17a and b) [22]. As exhibited in Fig. 17c and d, the cell with SiO_2_-IL-ClO_4_ displayed a more robust cycling stability compared to that without SiO_2_-IL-ClO_4_. Moreover, fluoroethylene carbonate (FEC) [83], trimethyl phosphate (TMP) [84] and indium triiodide (InI_3_) [85] with suitable content were also regarded as an excellent additive.

#### Ether-based electrolyte

Small-molecule and/or long-chain ethers have all been considered as promising electrolytes for Li-S batteries. On account of their high boiling/flash points, non-flammability and wide voltage window, long-chain ethers, such as TEGDME, have presented excellent electrochemical properties for Li-S batteries. However, the Na-S chemistry in TEGDME electrolytes exhibited low specific capacity (only ∼350–550 mAh g^–1^) and poor cycling stability [86]. The huge capacity loss may be caused by the high solubility of long-chain polysulfides and severe shuttling of sodium polysulfides in the TEGDME-based electrolyte. At the same time, it also failed to form a stable SEI film and suppress sodium dendrites on the anode side. The development of novel type ether-based electrolyte is an inevitable trend.

**Figure 18.**
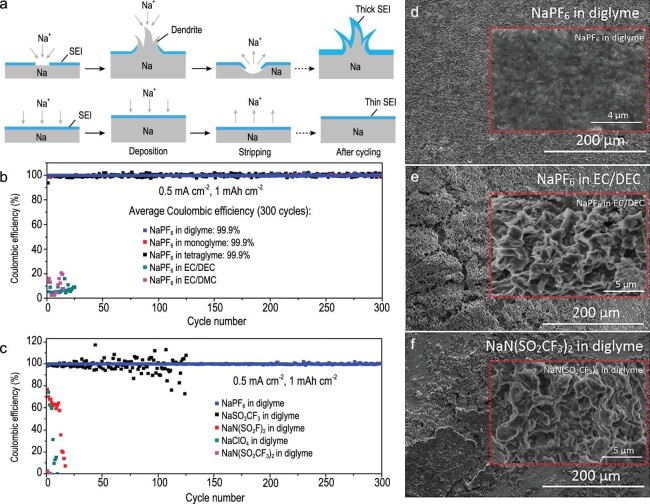
Regulation of Na plating/stripping by a novel type of electrolyte. (a) Comparison of smooth and rough Na plating/stripping process. (b and c) The average Coulombic efficiency of Na plating/stripping with 1 M NaPF_6_ in various electrolyte solvents and 1 M of various salts in diglyme, respectively. (d–f) SEM images of Na metal surface after a cycle in various electrolytes. Adapted with permission from [87]. Copyright 2015, American Chemical Society.

**Figure 19.**
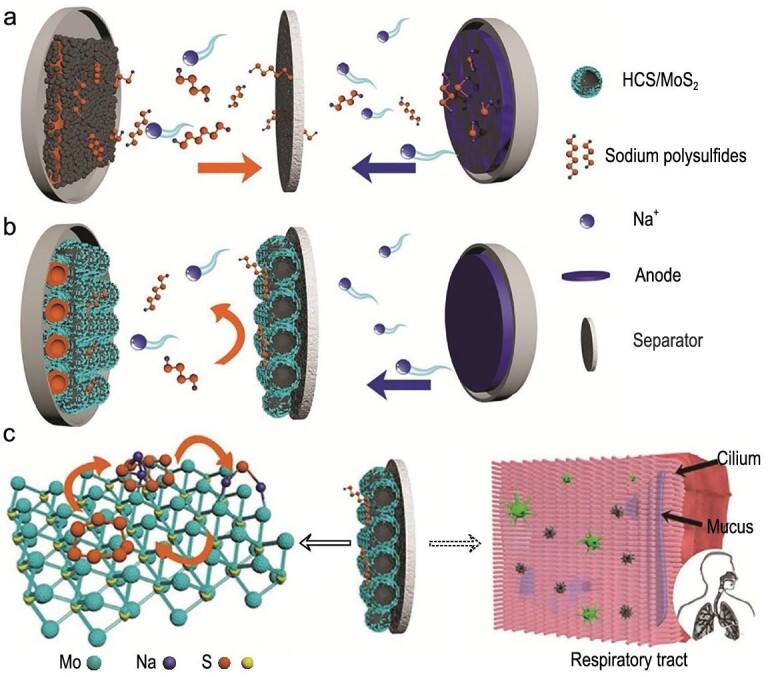
Surface (MoS_2_) modification of traditional GF separator. Schematic diagram of (a) the S/C composite and (b) the S@HCS/MoS_2_ electrode with modified GF during the discharge process. (c) Schematic diagram of the absorption and respiratory cilia. Adapted with permission from [90]. Copyright 2019, Wiley.

Cui’s group first reported the new ether-based electrolyte of NaPF_6_ in diglyme, monoglyme or tetraglyme [87]. The glyme-type electrolyte achieved a smooth Na plating/stripping with thinner SEI, leading to a highly reversible long-term cycle at room temperature (Fig. 18a). As shown in Fig. 19b and c, the average Coulombic efficiency close to 100% was obtained using 1 M NaPF_6_ in diglyme, higher than other electrolytes after 300 cycles. Unlike NaPF_6_ in EC/DEC and NaN(SO_2_CF_3_)_2_ in diglyme, NaPF_6_ in diglyme enabled a smooth surface with uniform SEI, which has high impermeability to electrolyte solvent and favorability to non-dendritic growth, verified by the SEM characterization (Fig. 18d–f). All the battery devices fabricated with a combination of non-dendritic Na electrodeposition and highly stable SEI at the Na-metal anode showed superior electrochemical performance.

## SEPARATOR

A separator in a battery is used to prevent electrical contact between the anode and cathode, as well as ensure effective ion transport back and forth. An ideal separator should feature good mechanical/thermal properties, wettability of the electrolyte, electrochemical stability, uniform dimension and cost-effectiveness. For sulfur-based batteries, the prevention of polysulfide shuttling is another important factor to consider for separator design. Traditionally, polypropylene (PP) and glass fiber (GF) separators are commonly used. Nevertheless, the micron-sized pores of the PP and GF separator cannot prevent the diffusion of sodium polysulfide. The design of membranes or separation materials has received considerable recent attention, including two main parallel strategies: surface modification of the traditional separator and development of a novel separator.

### Surface-modified traditional separator

Given the fact that traditional PP or GF separators show limited effectiveness in isolating sodium polysulfides, some research has focused on the modification of membranes by coating polar compounds on their surface. Bauer *et al.* reported a sodiated Nafion membrane coated conventional PP separator to achieve significant inhibition of sodium polysulfide shuttling [88]. The coated separator still has sufficient ionic conductivity, which could be confirmed by the unreduced discharge voltage value. Cengiz *et al.* proposed a GF separator with rich negatively charged sulfonic groups coated by an Al_2_O_3_-Nafion membrane, where Al_2_O_3_ worked as adsorbent for trapping polysulfide anions, while Nafion has high cation selectivity [89]. Compared with the untreated separator, the modified separator presented both higher reversible capacity and better cycling stability, suggesting higher sulfur utilization and more excellent isolation of polysulfides.

Recently, Yang *et al.* modified GF by uniformly coating MoS_2_ decorated hollow carbon spheres (HCS/MoS_2_) composite [90]. Figure 19a and b visually compares the behavior of two separators (non-modified and modified glass fiber), where the modified separator can effectively adsorb the polysulfides and prevent their shuttling, working as a defense system during the discharge process. The adsorption capacity of HCS/MoS_2_ was investigated by polysulfide adsorption test, ultraviolet-visible absorption and XPS. Interestingly, the efficacy of the HCS/MoS_2_-modified separator is similar to that of cilium in the human respiratory tract, which isolates harmful substances (Fig. 19c).

### Novel separator

The novel separator, as an alternative to the unqualified conventional separator, must be a remarkable ion selective membrane that possesses high ionic conductivity and superior capability to trap polysulfides. Various carbon materials (porous carbon, graphene), polymers (PVDF, Nafion) and inorganic materials (oxide, chalcogenides) have been proven as separators or additives in a separator for the isolation of lithium polysulfides. For instance, Lei *et al.* proposed a negatively charged graphene composite separator, which offered a ‘charge-repulsion’ approach to effectively suppress polysulfide shuttling [91]. These can provide a valuable reference for the design of membrane materials, although current research on a novel separator for RT-Na-S batteries is limited. So far, only the Nafion membrane [92], polybenzimidazole (PBI) membrane [93] and white graphite (*h*-BN) incorporated polymer membrane [94] were investigated as a separator for RT-Na-S batteries.

**Figure 20.**
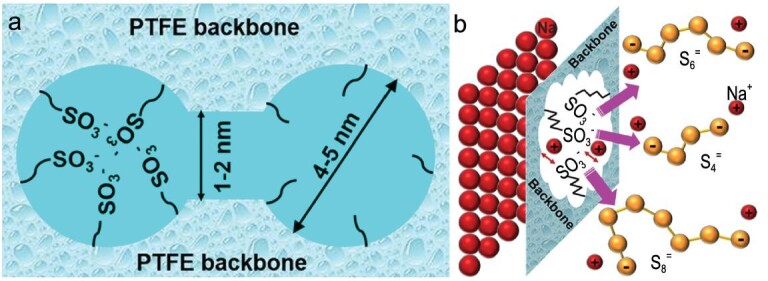
Composition and function of novel Nafion separator. (a) The architecture and (b) ionic selectivity of the Nafion membrane. Adapted with permission from [67]. Copyright 2016, American Chemical Society.

On a Nafion membrane, a hydrophobic polytetrafluoroethylene (PTFE) skeleton and a hydrophilic sulfonic acid groups cluster (∼4–5 nm) are connected to each other through a hydrophilic channel (∼1–2 nm), as shown in Fig. 20a [67]. The PTFE skeleton provides electrochemical stability, while -SO_3_^–^ provides a negatively charged chemical environment (Fig. 20b). Therefore, the Nafion membrane with polar surface can selectively shield polysulfides by ionic interactions, ensuring effective Na^+^ transport paths. By inhibiting the diffusion of polysulfides to the anode, the Nafion membrane can significantly increase the capacities and cycle retentions compared with the traditional separator.

Saroja *et al.* recently incorporated hexagonal white graphite (*h*-BN) into a polymer membrane, PVDF-HFP/poly(butyl methacrylate) (PBMA) blend [94]. By calculation, the ionic conductivity of a BN/polymer composite separator can be 10^–3^ S cm^–1^, an order of magnitude higher than that of a pure polymer separator. More importantly, the N and B sites in white graphite can be effective adsorption sites in trapping sodium polysulfides. The dual functions ultimately prompted significant enhancement of electrochemical performance (87.6% in specific capacity).

There have been some reports of separators, but these references are far from enough. And more strategies and more detailed mechanisms need to be proposed, combining the experience of Li-S electrochemistry. High ionic conductivity, excellent isolation ability for polysulfide compounds, and the presence of catalytic sites, etc., are all what the future multi-functional separator should possess. For soluble sodium polysulfides, is the ‘adsorption property’ or ‘repulsion ability’ of the separator worthier of attention? All of these still need to be further studied in subsequent studies.

## COLLECTOR

The current collector (as in the case of Cu and Al foil) mainly collects the current generated by the active material of the battery to form a larger current flux for external output. Therefore, the current collector should fully contact the active material, and the internal resistance should be as small as possible. More importantly, the current collector is also used as a substrate for alkaline metal plating/stripping in metal-based batteries, which has a vital influence on the plating/stripping behavior. For the Na metal battery, Al foil with lower cost and lighter weight shows advantages over Cu foil, because it is not alloyed with Na.

**Figure 21.**
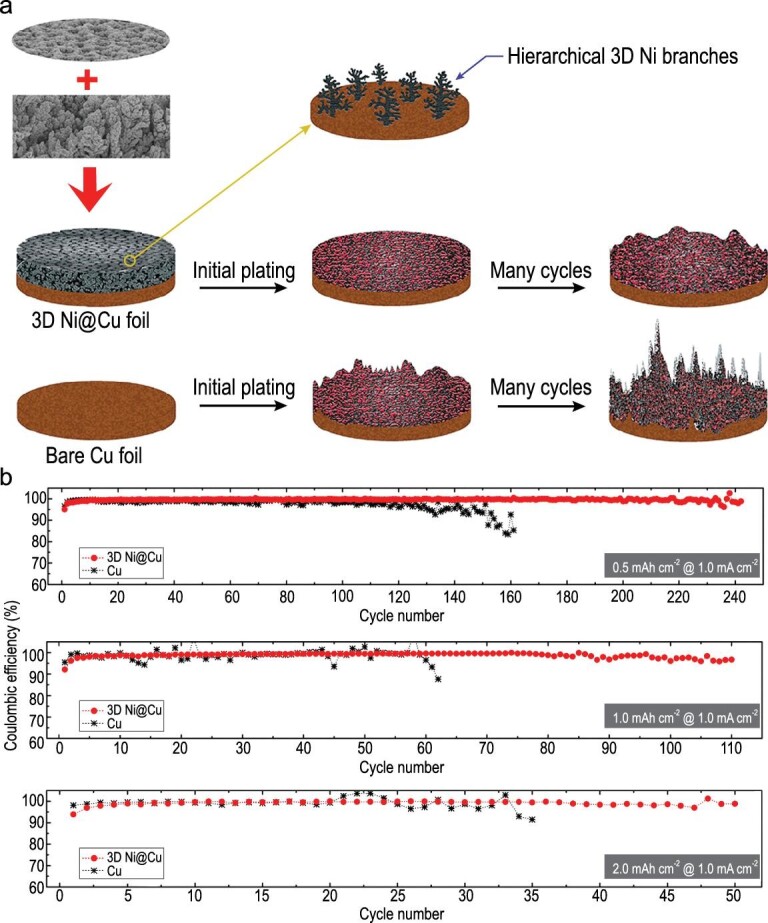
A 3D porous Ni electrodeposited Cu current collector. (a) Na plating/stripping process on a 3D Ni@Cu and bare Cu foil. (b) The Coulombic efficiencies of the 3D Ni@Cu and bare Cu foil during Na plating/stripping at 1.0 mA cm^–2^ under different areal loading of Na. Adapted with permission from [97]. Copyright 2017, Elsevier.

Cohn *et al.* proposed a carbon nucleation layer formed on the Al current collector to achieve stable plating/stripping of Na metal [95]. After *in situ* Na plating, the cell could maintain a low voltage hysteresis (∼45 mV) in the current range of 0.5–4 mA cm^–2^, obtain a high surface capacity (up to 12 mAh cm^–2^) with a Coulombic efficiency close to 100%, and display long durability (more than 1000 cycles at 0.5 mA cm^–2^). Then, a porous Al foil was designed by Liu *et al.* as the Na plating/stripping substrate to inhibit formation and growth of dendrites [96]. The interconnected porosities increased the available sites for Na nucleation, and adjusted the Na^+^ flux to achieve uniform charge distribution, resulting in uniform plating/stripping. Equipped with porous Al, the Na metal anode maintained a low voltage hysteresis for more than 1000 cycles. Compared with planar Al foil, the average Coulombic efficiency (above 99.9%) of porous Al foil increased during the plating/stripping process. Recently, Mulder *et al.* deposited a layer of honeycomb hierarchical 3D porous nickel on a traditional Cu current collector (3D Ni@Cu) through a one-step hydrogen bubble dynamic template (HBDT) electrodeposition method (Fig. 21a) [97]. As a current collector of Na metal batteries, 3D Ni@Cu exhibited excellent electrochemical behavior (Fig. 21b), delivering a stable and reversible Na plating/stripping process with Coulombic efficiencies close to 100% under varied areal capacities.

It can be seen that design of porous structure, surface treatment or coating or deposition are effective in inhibiting Na dendrite growth and stabilizing the plating/stripping process. Note that these strategies should be coupled with appropriate electrolytes and cathodes to further develop high-performance Na-based batteries. In addition, it is also particularly important to develop a new type of high conductivity and ultra-thin current collector to replace the traditional aluminum foil or copper foil, which is a promising way to enhance the energy density of the practical RT-Na-S battery.

## CELL CONFIGURATION

The conventional cell configuration of RT-Na-S batteries may be inappropriate due to the existence of the shuttling effect and the formation of Na dendrites. Inserting an interlayer in the original configuration is considered as an effective strategy for optimizing battery performance. On the cathode side, the interlayer should be able to capture soluble polysulfide species and limit their diffusion to the sodium anode. On the anode side, the interlayer can adjust the Na^+^ ion flux at the interface and inhibit the growth of dendrites. In addition, the conductivity of the interlayer is also required, which should enhance the conductivity of the entire electrode to improve the utilization of active materials. Therefore, various materials have been used as interlayers, such as micro/mesoporous carbon, CNFs/CNTs, porous metal foam, carbon paper and porous biomaterials.

**Figure 22.**
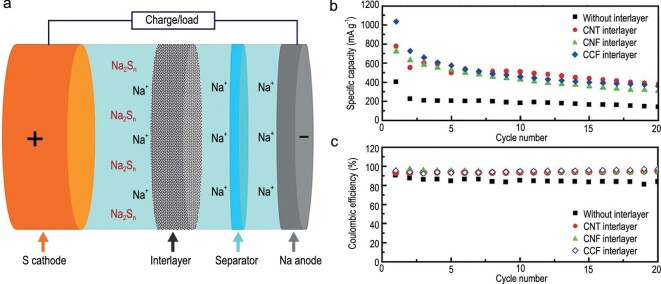
Inhibition of polysulfide shuttling by the interlayer on the S cathode side. (a) Cell configuration with an interlayer close to the S cathode. (b) The capacities and (c) Columbic efficiencies of the cell without and with various interlayers. Adapted with permission from [13]. Copyright 2014, Wiley.

When it comes to the prevention of the shuttling effect, Manthiram’s group proposed a cell configuration for the RT-Na-S battery with an interlayer close to the S cathode [98]. Later, the effects of three intermediate layers (CNF film, CNTs film and commercial carbon foam (CCF)) between the separator and S cathode on the shuttling of soluble polysulfides were investigated, as shown in Fig. 22a [13]. These conductive interlayers ensured a direct contact with the S cathode, which did not increase the interface impedance or affect the electrolyte penetration. Importantly, the interlayer can fix the soluble polysulfides on the side of the cathode through electrochemical deposition, fundamentally inhibiting their shuttling during the charging/discharging process. Comparing the electrochemical performance of batteries equipped with and without an interlayer, it is clearly found that the interlayer had an important contribution in improving capacity and Coulombic efficiency, as shown in Fig. 22b and c, respectively. The different interlayers with difference in wettability of electrolyte may lead to weak differences in electrochemical properties. For example, the first discharge voltage of a CNT-containing cell is relatively lower than that of a CNF-containing cell or CCF-containing cell.

**Figure 23.**
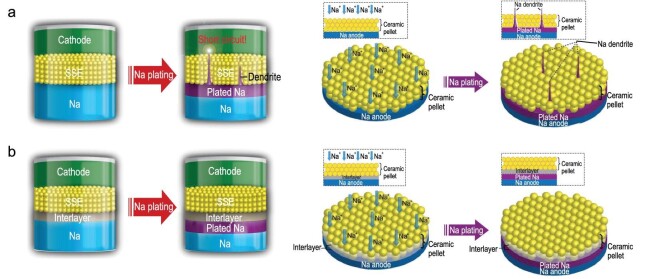
Suppression of dendrites by the interlayer on the Na anode side. Schematic configurations of solid-state cells equipped (a) without an artificial interlayer and (b) with an artificial interlayer, and their corresponding Na plating process. Adapted with permission from [99]. Copyright 2017, American Chemical Society.

When it comes to the suppression of Na dendrites, Goodenough and his co-workers reported a thin dry polymer (cross-linked poly(ethylene glycol) methyl ether acrylate, CPMEA) film as interlayer, as shown in Fig. 23 [99]. Inserting a polymer interlayer reduced the (solid–solid) interface resistance between anode and electrolyte, and realized the reversible Na plating/stripping without dendrites growth in the anode. In addition, the interlayer had better wettability to sodium, providing a uniform ceramic/interlayer/sodium interface to achieve a uniform sodium flux across the interface. Although this structure was used for a rechargeable sodium all-solid-state battery, the strategy to suppress dendrite formation and growth could be followed in RT-Na-S batteries.

## CONCLUSIONS AND PERSPECTIVES

With similar electrochemistry to Li-S batteries, RT-Na-S batteries have attracted increasing attention for grid-scale energy storage, with the potential benefit of having high capacity, rich abundance, low cost and environmental benignity. However, RT-Na-S batteries still suffer from a number of persistent challenges, including poor electrical conductivity of sulfur and the discharge products (Na_2_S_2_ and Na_2_S), large volume change of the cathodes, polysulfide shuttling effect, and formation and growth of Na dendrites. This review discusses opportunities and critical challenges for RT-Na-S batteries, and summarizes the latest advances in electrode materials, electrolytes, separators and cell configurations with regard to RT-Na-S batteries.

A diverse set of strategies have been explored to address these critical challenges. To combat poor electrical conductivity and the polysulfide shuttling effect, the encapsulation of sulfur or long-chain polysulfides into a 3D conductive matrix (e.g. porous carbonaceous materials or MOFs) represents an attractive strategy. Additionally, the formation of covalent bonding between polysulfide species with hosting matrix through S−C and S-polymer, S-MXene and S-metal/metal bonds may also help mitigate the polysulfide shuttling effect. To inhibit Na dendrite growth during cycling, strategies including constructing various structured sodium and coating artificial protective layers on metallic sodium have been developed. Additionally, the optimization of electrolyte composition/additives may help in suppressing the dissolution of polysulfides and at the same time ensure uniform SEI formation on the Na metal surface to ensure stable Na plating/stripping and minimize Na dendrite formation. Lastly, the modification of the separator and improvement of cell configuration also represent an attractive engineering approach for retarding the polysulfide shuttling effect while allowing efficient Na^+^ transport to ensure uniform Na deposition.

Despite the increased effort in each one of these aspects, a comprehensive solution that can simultaneously address these challenges is lacking. It is not unusual that an improvement in one challenge is achieved at the sacrifice of the other problems. To realize high-performance RT-Na-S batteries, the concerted developments in these critical aspects are essential. To this end, a more systematic, mechanistic understanding of complex polysulfide conversion, polysulfide shuttling, sodium deposition/stripping and Na^+^ transport process is essential for developing the comprehensive strategies that can fundamentally address these problems and turn RT-Na-S batteries into a real technology. In this regard, considering the close similarity to the Li-S batteries and rapid development in this field, the knowledge developed in Li-S batteries should be leveraged for the development of RT-Na-S batteries. To this end, a number of key challenges should be closely considered.

The polysulfide shuttling effect is fundamentally originated from the slow polysulfide conversion process that leads to accumulation of soluble polysulfides in the electrolyte solution and worsening shuttling effect. To fundamentally address this challenge, rational design and synthesis of electrocatalysts (e.g. heteroatom doped carbon matrix) and incorporation of such catalysts in the S host could accelerate polysulfide conversion and fundamentally mitigate polysulfide dissolution and shuttling effect [65]. Additionally, considering the solubility of polysulfides in the electrolyte, systematic screening and evaluation of a novel electrolyte may prove beneficial. Additionally, a proper interfacial interlayer coating on the separator with large adsorption energies towards long-chain sodium polysulfides could also help retard the polysulfide shuttling effect to enhance the cycling stability [91]. The Na dendrite formation fundamentally originates from inefficient Na^+^ transport and Na^+^ concentration polarization that together lead to non-uniform sodium deposition. To this end, the proper design of separators as well as SEI layers that can allow efficient and uniform Na^+^ transport could offer a fundamental solution. In that regard, the unconventional separator design or solid-state electrolyte may offer exciting opportunities to simultaneously hinder the polysulfide shuttling effect while unifying Na^+^ transport flux. Lastly, for practical batteries, high sulfur content and high areal mass loading is essential for boosting the overall device-level energy density. To this end, continued effort in the design and development of 3D conductive scaffolds and the efficient incorporation of nanoscale S cathode material into such a conductive scaffold is essential for retaining specific capacity without sacrifice in rate capability [100].
